# Nanoarchitectonics for Pentagon Defects in Carbon: Properties and Catalytic Role in Oxygen Reduction Reaction

**DOI:** 10.1002/smtd.202500069

**Published:** 2025-04-22

**Authors:** Guoping Chen, Taro Koide, Junji Nakamura, Katsuhiko Ariga

**Affiliations:** ^1^ International Institute for Carbon‐Neutral Energy Research (I2CNER) Kyushu University 744 Motooka, Nishi‐ku Fukuoka‐shi Fukuoka 819‐0395 Japan; ^2^ Department of Chemistry and Biochemistry Graduate School of Engineering Kyushu University Moto‐oka 744 Nishi‐ku Fukuoka 819‐0395 Japan; ^3^ Department of Advanced Materials Science Graduate School of Frontier Sciences The University of Tokyo 5‐1‐5 Kashiwanoha Kashiwa Chiba 277‐8561 Japan; ^4^ Research Center for Materials Nanoarchitectonics National Institute for Materials Science Namiki 1‐1 Tsukuba 305‐0044 Japan

**Keywords:** carbon, oxygen reduction reaction, pentagon

## Abstract

The oxygen reduction reaction (ORR) is a crucial process in electrochemical energy technologies, featuring fuel cells and metal‐air batteries in the coming carbon‐neutral society. Carbon materials have garnered significant attention as economical, sustainable alternatives to precious metal catalysts. In particular, there have been increasing reports recently that pentagons introduced into graphitic carbons promote catalytic activity for ORR. In addition, interesting studies are reported on carbon materials’ synthesis, characterization, and spin polarization properties with pentagonal defects. This review comprehensively summarizes the formation mechanism, characterization, spin, oxygen (O_2_) adsorption, and ORR catalytic activity of carbon catalysts with pentagonal defects. By connecting the dots between theoretical insights and experimental results, this review elucidates the fundamental principles governing pentagon‐related activity and offers perspectives on future directions for designing efficient ORR catalysts based on carbon materials.

## Graphical abstract

“Pentagon” in carbon framework is focused in this review, although hexagon is standard atomic frames in carbon materials. This review elucidates the fundamental principles governing pentagon‐related activity and offers perspectives on future directions for designing efficient oxygen reduction reaction catalysts based on carbon materials. Nanoarchitectonics for pentagon defects will be a key step in materials science for energy.

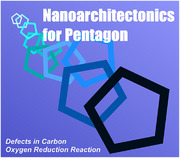

## 1 Introduction

### 1.1 Importance of Nanostructure Control, Nanoarchitectonics

Humanity is on an incredible mission to develop science and technology every day to tackle the challenges of our time, for energy,^[^ ^1^ ^]^ environment,^[^ ^2^ ^]^ and biomedical issues.^[^ ^3^ ^]^ Materials science is the key to this progress. The development of materials with excellent functions according to need is an exciting area of research. Thanks to the development of the basic fields of material science in the 20th century, we have succeeded in creating various materials. Then, in the second half of the 20th century, the concept of nanotechnology was initiated,^[^ ^4^ ^]^ which opened the door to observing^[^ ^5^ ^]^ and manipulating^[^ ^6^ ^]^ atoms and molecules, and analyzing their properties with nano‐level understanding.^[^ ^7^ ^]^ This has led to the exciting realization that the functionality of materials depends heavily on their nanostructures.^[^ ^8^ ^]^ Indeed, material science and nanotechnology are now the two wheels of functional material development. In the 21st century, these fields have now come together under a new and novel concept called nanoarchitectonics,^[^ ^9^ ^]^ which represents the next step in the evolution of nanotechnology.^[^ ^10^ ^]^ This new concept involves architecting functional materials from atoms, molecules, and nanomaterials using insight into nanotechnology.

It is clear that, given the advances in science and technology, controlling the nanostructure is often more important than developing the material itself to achieve more sophisticated functions. Take carbon‐based materials, for instance. The success of nanoporous carbon is a prime example of nanoscale structure control. Compared to conventional carbon materials, nanoporous carbon materials show excellent properties in catalysis^[^ ^11^ ^]^ and pollutant removal^[^ ^12^ ^]^ upon controlling the enhanced surface area and regulated nanospace. A more distinct development is the explosive growth of various nanocarbons. Even materials made of the same carbon, such as fullerenes,^[^ ^13^ ^]^ carbon nanotubes,^[^ ^14^ ^]^ and graphene,^[^ ^15^ ^]^ show completely different electronic properties if their dimensionality and quantum structure are different. The development of carbon nanoarchitectonics, which is even more sophisticated, is currently blooming. The key to this is structural control at the atomic skeleton level of hexagonal and pentagonal structures, as the internal structures of nanocarbons. As this paper proves later, carbon materials containing pentagon structures exhibit extremely distinctive physical and chemical properties compared to those consisting only of hexagon structures. Carbon materials are also pivotal in the development of energy materials that do not use precious metals.^[^ ^16^ ^]^ Cutting‐edge research is focused on investigating how skeletal control affects energy‐related functions.

**Figure 1:**
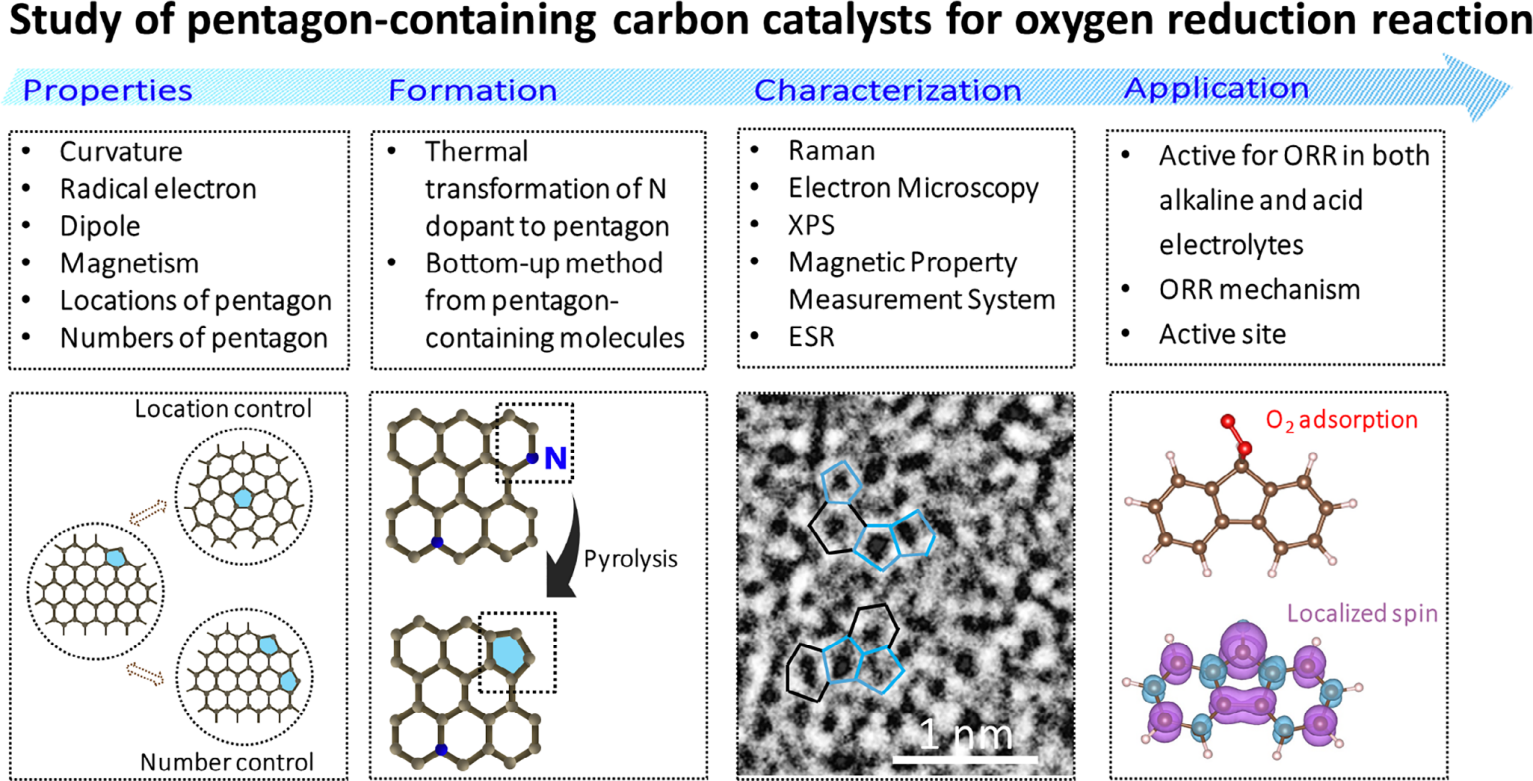
Schematic illustration of pentagon defect in carbon for oxygen reduction reaction.

In this review paper, as shown in **Figure** **1**, we will consider the role of the pentagon structure in the oxygen reduction reaction (ORR), one of the key components in energy applications. Besides, we will explore the formation mechanisms of pentagon defects in carbon materials, from high‐temperature annealing to bottom‐up synthesis strategies. It will also examine their structural characteristics, as revealed by state‐of‐the‐art characterization tools, and discuss their impact on ORR activity in both alkaline and acidic environments. By digging into the unique properties of pentagon defects, this review aims to provide insights into their pivotal role in advancing ORR catalysis and their potential to bridge the gap between theoretical predictions and experimental realizations.

### 1.2 Basics of Oxygen Reduction Reaction

The ORR is a fundamental electrochemical process central to the operation of energy transformation and conservation technologies, particularly in systems like fuel cells and metal‐air batteries.^[^ ^17^ ^]^ It is therefore one of the most actively researched topics in electrochemistry and catalytic chemistry. The ORR occurs at the cathode, where O₂ undergoes a reduction reaction, transforming it into water (in acidic electrolytes) or hydroxide ions (in alkaline electrolytes). This process not only dictates the device efficiency but also influences key parameters such as power density, durability, and cost‐effectiveness, making ORR catalysis a crucial area of research for clean energy advancements. However, the ORR is inherently sluggish, primarily due to its complex multi‐electron transfer mechanism and the strong O═O bond in O_2_, which requires substantial activation energy to break. This slow reaction rate limits the overall performance of devices, necessitating highly active catalysts to reduce overpotentials and accelerate the reaction.^[^ ^18^ ^]^

Platinum‐based catalysts have long been effective for the ORR, but the high cost and limited stability of platinum in acidic environments have spurred the search for alternative, more sustainable catalysts.^[^ ^19^ ^]^ Among these, carbon‐based materials hold the most promise as resistant to corrosion and durable electrocatalysts in strong acid electrolytes. In addition, they have the advantages of being abundant, affordable, and having tunable electronic properties. The versatile hybridization of carbon enables it to form a variety of allotropes, including fullerenes, carbon nanotubes (CNTs), and graphene. The landmark exploration in 2009 of nitrogen (N)‐doped CNTs as efficient ORR electrocatalysts revolutionized the field, drawing attention to the significance of carbon‐based materials for catalysis.^[^ ^20^ ^]^ Since then, understanding the transformation of inactive carbon (C) atoms into active catalytic sites through doping has become a key area of research. Significant efforts have been directed toward elucidating the functions of the three primary N compositions: graphitic‐N, pyridinic‐N, and pyrrolic‐N.^[^ ^17^ ^]^ This research focus later expanded to include other dopants and, eventually, defect‐rich carbon materials without dopants, signaling a shift from dopant introduction to defect optimization. Extensive experimental research and density functional theory (DFT) calculations have indicated that the activation of carbon stems from the uneven redistribution of charge and spin densities in C atoms near both dopants and defects.^[^ ^19a^ ^]^

ORR can proceed via two primary pathways: the four‐ and two‐electron pathways.^[^ ^18^ ^]^ The four‐electron pathway is considered the most desired mechanism in developing new green energy devices because of its high efficiency. O_2_ is directly reduced to water or hydroxide ions in the four‐electron pathway through a four‐electron transfer. The reaction steps are as follows:

(1) $$O_{2}+2H_{2}O+4e^{-}\to 4OH^{-}\;\left(\mathrm{alkaline}\;\mathrm{electrolyte}\right)$$

(2) $$O_{2}+4H^{+}+4e^{-}\to 2H_{2}O\;\left(\mathrm{acid}\;\mathrm{electrolyte}\right)$$

In an alternative pathway, O_2_ is reduced through a two‐electron transfer to form hydrogen peroxide (H_2_O_2_), which is the target reaction for producing green oxidant and a significant chemical product. However, hydrogen peroxide is highly reactive, potentially causing the degradation of carbon catalysts and thereby compromising their durability. The reaction steps are as follows:

(3) $$O_{2}+H_{2}O+2e^{-}\to \mathrm{HO}O^{-}+OH^{-}\;\left(\mathrm{alkaline}\;\mathrm{electrolyte}\right)$$

(4) $$O_{2}+2H^{+}+2e^{-}\to H_{2}O_{2}\;\left(\mathrm{acid}\;\mathrm{electrolyte}\right)$$

The peroxide intermediate can then be further reduced to water or hydroxide ions. Still, this step tends to be less efficient and produces reactive intermediates compared to the direct four‐electron pathway. The reaction steps are as follows:

(5) $$\mathrm{HO}O^{-}+H_{2}O+2e^{-}\to 3OH^{-}\;\left(\mathrm{alkaline}\;\mathrm{electrolyte}\right)$$

(6) $$H_{2}O_{2}+2H^{+}+2e^{-}\to 2H_{2}O\;\left(\mathrm{acid}\;\mathrm{electrolyte}\right)$$

It is not clear what factors in catalytic performance determine the two‐ and four‐electron reduction mechanisms. It has been reported that the distinction between two‐ and four‐electron pathways arises from the varying types of O_2_ adsorption, which can initiate distinct reaction mechanisms due to the binding strength between O_2_ and the active site.^[^ ^21^ ^]^

The spin properties of the catalyst are thought to be important in the adsorption of O_2_, which is the first elementary step of the ORR. The preservation of total angular momentum in chemical reactions is an essential principle.^[^ ^22^ ^]^ This principle enforces electron and nuclear spin selectivity in reactions: only reactant spin states that match the total spin of the products are chemically active. In other words, reactions engaging in reactants and products with differing spin quantum numbers are theoretically forbidden or require additional energy to induce a spin flip.^[^ ^22^ ^]^ The O_2_ molecule exemplifies this concept, with its two unpaired electrons in parallel spins on the π* orbitals, resulting in a triplet ground state. In contrast, OH⁻ and H_2_O exist in singlet states.^[^ ^23^ ^]^ Spin catalysis refers to a phenomenon where chemical reactions are facilitated by substances that either overcome spin prohibition or lower the activation barrier via spin uncoupling triggered by a paramagnetic catalyst. More simply, a reacting particle can exchange its magnetic moment with a “spin catalyst”—a substance possessing a pseudo‐spin‐degenerate ground state or low‐lying excited states—enabling the desired spin state while conserving the overall spin of the system.^[^ ^24^ ^]^ In the context of ORR, the catalyst's interaction with O_2_ species plays a critical role. The catalyst supplies the additional energy and spin electrons needed for spin state transitions. However, the conversion between singlet OH^−^/H_2_O and triplet O_2_ requires three electrons aligned in the same spin direction.^[^ ^25^ ^]^ If the catalyst cannot effectively pass three spin‐down and one spin‐up electrons, certain electrons must flip spins, demanding extra energy during the ORR process.^[^ ^23^ ^]^ Therefore, a comprehensive understanding of electronic spin behavior in electrocatalytic reactions is essential for unraveling the microscopic mechanisms governing ORR and for designing high‐performance, nonprecious metal catalysts.^[^ ^26^ ^]^ Many studies have focused on the interplay between electron spin states and ORR performance, emphasizing its importance in advancing catalyst design.^[^ ^26^ ^]^

### 1.3 Recent Progress toward Spin Science

**Figure 2:**
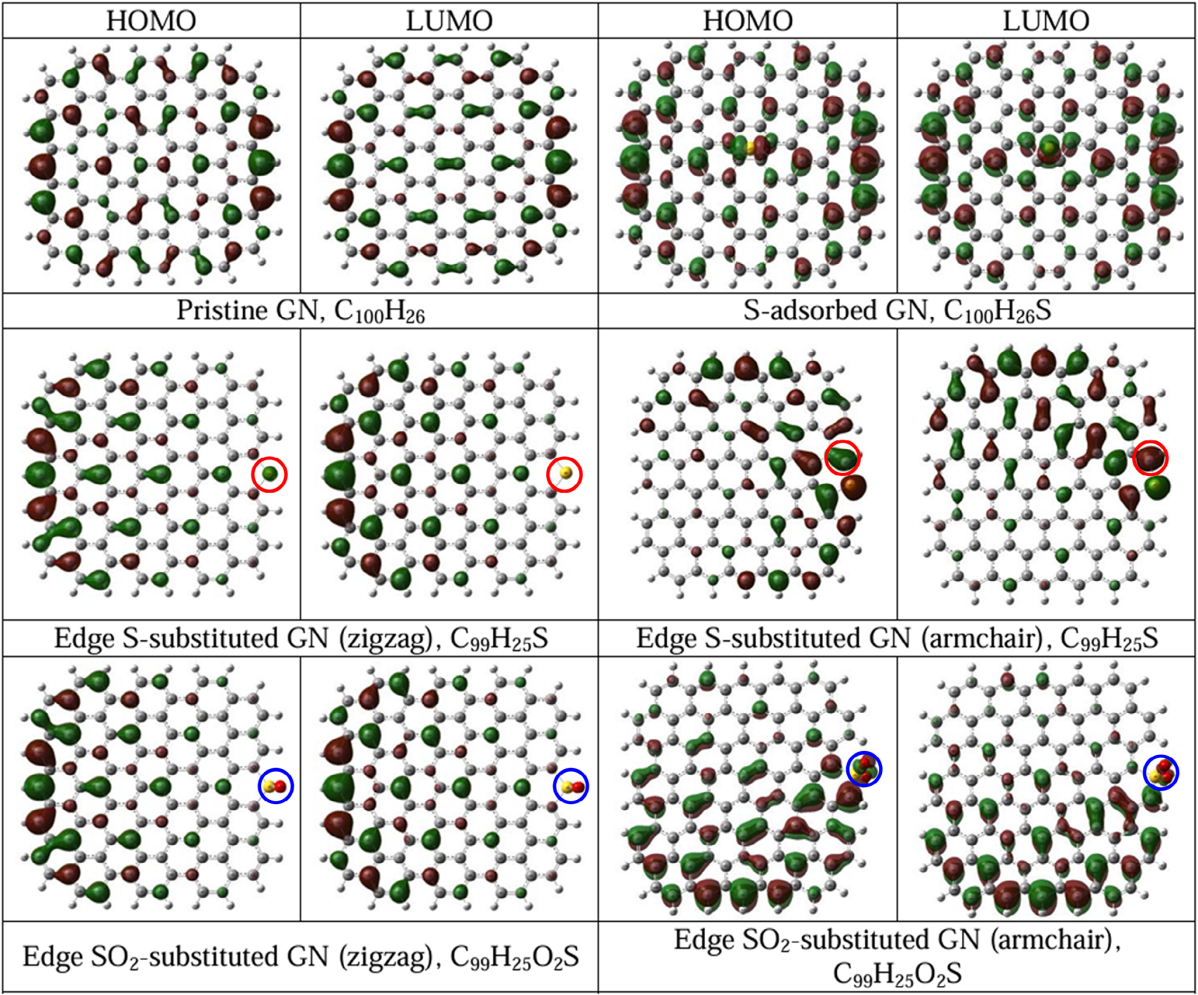
HOMO and LUMO distribution on the pristine graphene and sulfur‐doped graphene nanoplatelets (GN). Reproduced with permission.^[^ ^27^ ^]^ Copyright 2013, Wiley‐VCH.

Dai and Baek's group has reported that in addition to the commonly believed charge density, high electron spin density plays a significant role in the ORR performance of carbon catalysts. This group designed and synthesized sulfur‐doped graphene nanoplatelets (SGnPs) to investigate the electron spin effect in enhancing ORR activity.^[^ ^27^ ^]^ The edge‐selective sulfurization of graphene nanoplatelets was achieved via ball‐milling pristine graphite with sulfur. As shown in **Figure** **2**, spin‐polarized DFT simulations were employed to examine the role of spin polarization in Pristine GN, S‐adsorbed GN, edge‐substituted GN, and edge SO_2_‐substituted GN. In pristine graphene, the HOMO and LUMO orbitals are symmetrically distributed. However, they exhibit strong polarization when sulfur atoms are covalently bonded at the graphene edges (e.g., S or SO_2_ substitutions, highlighted in the circle). These polarized regions are likely to serve as active sites for the ORR. The findings further confirm that only edge‐sulfurized graphene demonstrates high catalytic activity while sulfur‐adsorbed one shows only a slight polarization and low catalytic activity. Compared to undoped graphene, SGnPs show significantly enhanced catalytic performance for ORR. Moreover, oxidized SGnPs (SOGnPs) exhibit superior magnetic moments among all S‐doped structures, correlating with their superior ORR activity. These results underscore that oxidized SOGnPs achieve further improvements in ORR efficiency.

**Figure 3:**
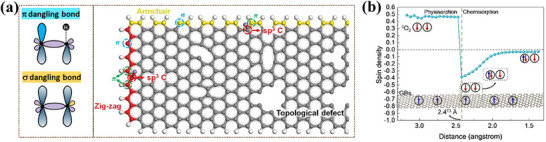
a) Molecular orbital diagram of σ and π dangling bond (left) and schematic diagram of typical defects in GB (right). b) The relationship between the spin density and the distance of O_2_ to the catalyst surface. The spin direction +1/2 is based on the GBs with high spin concentration; when the ground‐state O_2_ approaches a GB catalyst with high spin concentration, the spin direction of the GBs···O_2_ pairs turns antiparallel due to one antiparallel electron of the GBs occupying one of the O_2_ antibonding πg‐MO (π*) orbitals, thus leaving one antiparallel unpaired electron in π*. Reproduced with permission.^[^ ^28^ ^]^ Copyright 2023, Wiley‐VCH.

Cheetham group reported that radical electrons originating from defects on graphene belts (GBs) promote ORR.^[^ ^28^ ^]^ GB samples were prepared by annealing the samples at a series of temperatures from 700 to 1000 °C in air. They clarified that two radical species are generated on GBs with different origins: one is a σ‐radical created by the cleavage of C─C bonds, which tends to localize on carbon, and the other is a π‐radical created by the cleavage of C─H or C─O bonds, which tends to delocalize on the GB plane. (**Figure** **3**a) The difference between these radicals was observed in the ESR spectra, where the σ‐radicals were observed as narrow spectrums and the π‐radicals as broad spectrums. Theoretical calculations reveal that the spin density of defective graphene decreases due to chemisorption when O_2_ approaches closer than 2.473 Å to the surface. At this distance, electrons occupy one of the two antibonding π_g_ vacancies of O_2_ (Figure 3b). The catalytic activity of various GB samples was evaluated using linear sweep voltammetry (LSV) in an O_2_‐saturated 0.1 m KOH solution. Results demonstrated that catalytic performance is influenced by annealing temperature, with the GB‐900 sample exhibiting the best catalytic performance, achieving an onset potential (*E*_onset_) of 0.88 V. A detailed analysis of the correlation between catalytic activity and multiple parameters revealed that the strongest correlation was with the electron spin‐spin relaxation time (T_2_) of the narrow component (associated with σ radical concentration) in the ESR spectrum. Other factors, such as high conductivity, large surface area, high spin concentration, and the T_2_ of the broad component (linked to π radical concentration), did not show significant correlations. Furthermore, it has been reported that nonbonding edge states are present exclusively in the GBs’ zigzag‐edge regions, with no such states observed in the armchair‐edge regions.

**Figure 4:**
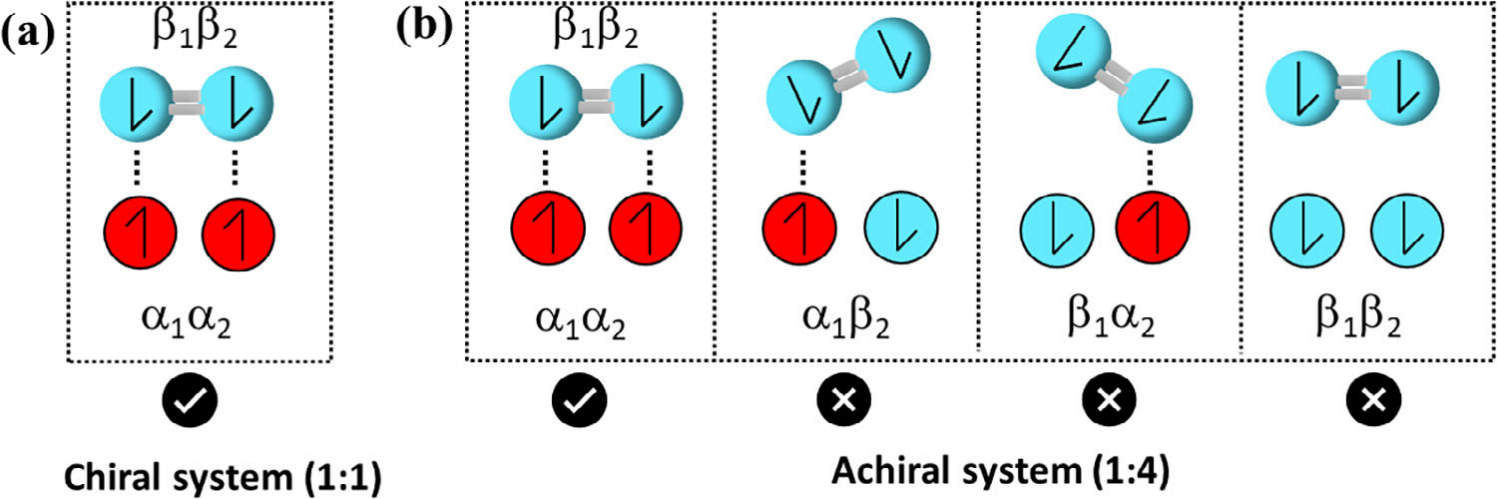
The possible spin states in the case of a) a chiral system, there is only one possible configuration in the chiral system, and the electrons are strongly coupled to the molecular frame. As a result, this is the only configuration that can lead to the reaction, and b) an achiral system, the two electrons can have four possible configurations from which only one of them leads to the reaction.^[^ ^30^ ^]^

In recent years, researchers have also explored the spin‐related properties of carbon materials, aiming to leverage phenomena like chiral‐induced spin selectivity (CISS) to enhance ORR. The CISS effect refers to a phenomenon of the preferential transmission of electrons with a specific spin orientation through chiral molecules. That means the chiral molecules act as spin filters, allowing electrons with a specific spin orientation to pass through more selectively than those with the opposite spin orientation.^[^ ^29^ ^]^ By controlling spin polarization, carbon‐based materials can promote more efficient O_2_ reduction by selectively allowing electrons with specific spin orientations to participate, thereby reducing energy barriers and enhancing reaction rates. This spin‐dependent catalysis, particularly in the context of pentagon‐rich structures, is a frontier in the field, offering a novel approach to optimizing ORR performance in carbon materials. Naaman group recently demonstrated that chiral electrodes achieve significantly higher current densities and lower overpotentials compared to nonchiral ones in catalyzing the ORR.^[^ ^30^ ^]^ This enhancement is attributed to the alignment of O_2_’s spin state with the chiral film, which reduces the entropic contributions to the free energy barrier (**Figure** **4**a,b). In achiral films, there are four potential spin states within the monolayer—αα, ββ, αβ, and βα—yet only one of these states enables efficient electron transfer to O_2_. As a result, the probability of reaction is restricted to one out of four. In addition, because the spins in achiral monolayers are not linked to the molecular frame, they do not cause a splitting of the spin states of O_2_, leaving the enthalpic barrier unchanged. LSV results demonstrate the impact of surface modification with a self‐assembled monolayer (SAM) of either achiral 3‐mercaptopropionic acid or chiral l‐cysteine. Despite their structural and length similarities, the chiral l‐cysteine SAM induces a notable shift of ≈0.17 V in the *E*_onset_ for ORR.

Advancing our understanding and optimization of the ORR through catalytic and spin‐dependent approaches remains crucial for the advancement of sustainable energy technologies, as efficient ORR catalysis is crucial in improving the economic and environmental sustainability of energy conversion and storage systems.^[^ ^31^ ^]^ The quest for productive and sustainable catalysts for the ORR has driven significant research into the properties and defect engineering of carbon materials.^[^ ^32^ ^]^ Among the myriad of defect types, the pentagon defect stands out as a particularly intriguing structural motif. Unlike the regular hexagonal arrangement in ideal sp^2^‐hybridized carbon, pentagonal defects disrupt the planar geometry, introducing local curvature and strain. These geometric distortions alter the electronic structure of the material, leading to localized charge redistribution and spin polarization, which are critical for catalysis. The presence of pentagonal defects not only enhances the adsorption of O_2_ but also facilitates the activation of O_2_ by creating energetically favorable sites for electron transfer. Recent studies combining DFT calculations and advanced spectroscopic techniques have confirmed that these defects act as intrinsic catalytic sites, outperforming conventional carbon structures, making them a promising avenue for the advancement of cost‐effective, metal‐free catalysts.

## 2 Pentagon in Carbon Materials

### 2.1 Curvature, Dipole, and Magnetism

Graphene, recognized as a truly 2D material, exhibits unique lattice defects characterized by reconstructed atomic arrangements that are unparalleled in other materials. This exceptional behavior is partially attributed to the diverse hybridization states of carbon, which allow for varying numbers of nearest neighbors and lead to the emergence of various stable structures, including carbyne, graphite, and diamond. In addition, sp^2^‐hybridized C atoms are capable of forming a range of polygons beyond the conventional hexagonal configuration, facilitating the development of distinct structures. Non‐hexagonal rings, such as those comprising five, seven, or eight members, can induce curvature in the graphene sheet or maintain a flat configuration, contingent upon adherence to specific symmetry rules. These non‐benzenoid rings are recognized as key structural motifs for modulating graphene's electronic, magnetic, and mechanical properties.^[^ ^33^ ^]^ Atomic network reconstructions facilitate the creation of a coherent defective lattice while avoiding the presence of under‐coordinated atoms. These reconstructed defects lack dangling bonds but exhibit localized reactivity due to the π and π* orbitals of sp^2^ C atoms. This localized reactivity facilitates the adsorption of other atoms onto the graphene layers, enhancing its functional versatility.^[^ ^34^ ^]^

**Figure 5:**
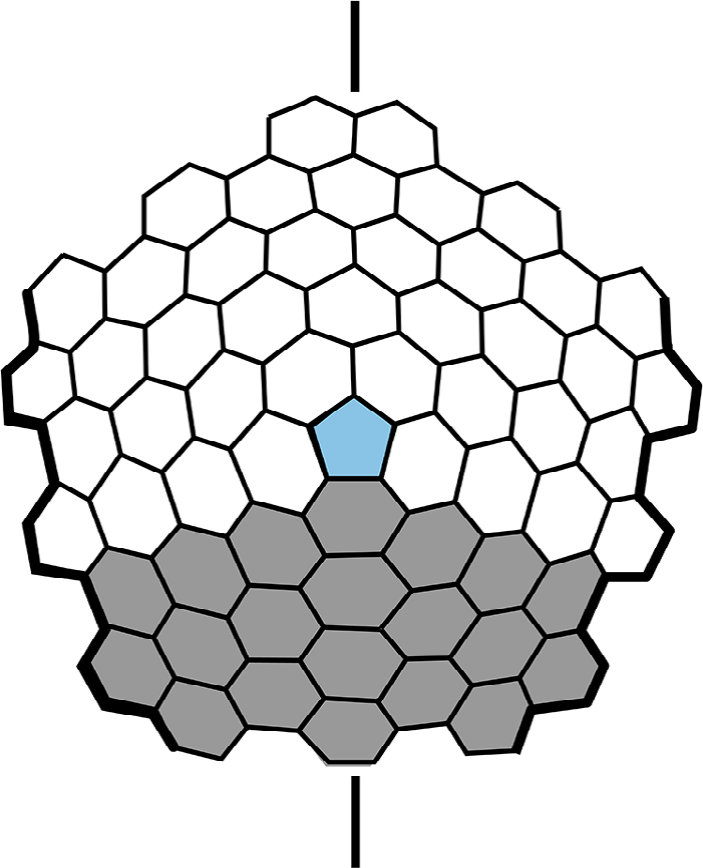
A +60° disclination produced by introducing a pentagon into a hexagon network. Reproduced with permission.^[^ ^35^ ^]^ Copyright 1992, Springer‐Nature.

In an ideal sp^2^‐conjugated carbon material, the hexagonal lattice would remain perfectly flat, with all pz orbitals aligned perpendicularly to the plane of the structure. However, this ideal configuration is rarely observed in real carbon materials. Topological defects, such as pentagonal rings, disrupt the pristine hexagonal arrangement.^[^ ^19a^ ^]^ Compared with a flat surface, the local 3D curved structure can induce more strained bonds arising from obvious geometrical bending. The distinct strain effect has the potential to influence the electronic configuration of active sites, as well as the adsorption and desorption dynamics of reaction intermediates, ultimately resulting in a significant improvement in catalytic efficiency. As shown in **Figure** **5**, it was reported that a surface containing a pentagon is curved positively.^[^ ^35^ ^]^

**Figure 6:**
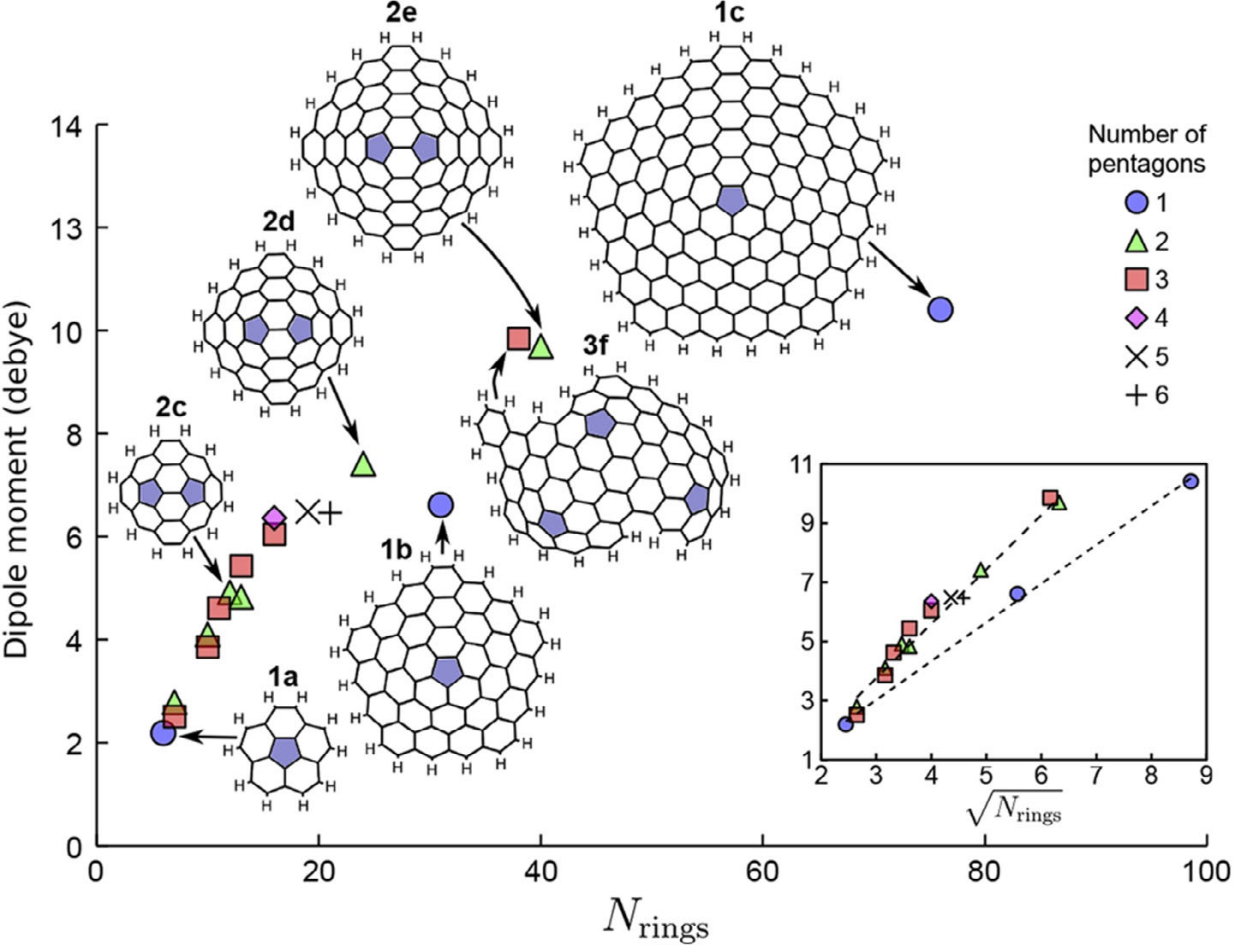
Dipole moment of curved polycyclic aromatic hydrocarbon molecules as a function of the number of rings. Inset: Dipole moment as a function of size comparing different numbers of pentagons. Reproduced with permission.^[^ ^38^ ^]^ Copyright 2017, American Chemical Society.

The incorporation of curvature into a hexagonal carbon lattice via a pentagonal point defect results in the generation of a substantial molecular dipole moment. For instance, corannulene has been experimentally measured to exhibit a dipole moment of 2.071 D, comparable to that of water (1.85 D).^[^ ^36^ ^]^ Nanometer‐sized curved arenes, referred to as nanocones, possess curved nuclei that extend into graphitic cones, incorporating a defined number of pentagonal structures at their apexes. Electronic structure calculations of nanocones ranging from 0.5 to 4 nm in size reveal substantial dipole moments between 10 and 35 D.^[^ ^37^ ^]^ The dipole moments observed in curved arenes can be ascribed to various contributing factors: (i) a flexoelectric dipole induced by curvature, arising from the polarization of π bonds perpendicular to the carbon framework, (ii) the angular orientation of C─H bonds, (iii) the transfer of charge from delocalized π‐electrons situated in hexagonal configurations to localized states at pentagonal sites, and (iv) the transfer of charge to localized states located at the periphery of the polycyclic aromatic hydrocarbon. To evaluate the relative importance of these factors, the Kraft group investigated the correlation between dipole moment and the molecular size as well as the number of pentagons.^[^ ^38^ ^]^ **Figure** **6** presents a detailed representation of the dipole moment in relation to the number of rings (*N*_rings_). For nanocone fragments, the dipole moment exhibits a linear dependency on the aromatic network's diameter. A similar linear trend is observed when plotting the dipole moment against the $\sqrt{N_{\mathrm{rings}}}$, which is proportional to the fragment's linear diameter (Figure 6, inset). The dipole moment exhibits an increase with the addition of pentagonal structures in polycyclic aromatic hydrocarbons; however, this increase plateaus after the incorporation of two or three pentagonal rings. As a result, for curved polycyclic aromatic hydrocarbons containing more than two pentagons, the dipole moment is predominantly determined by size rather than the number of pentagonal units.

Magnetism is relatively rare among the light p‐block elements in the second period of the periodic table, despite the ability of carbon to form a wide array of complex molecular structures. While pristine graphene is fundamentally nonmagnetic, numerous derivative materials and nanostructures, both experimentally developed and theoretically investigated, demonstrate a range of magnetic properties.^[^ ^39^ ^]^ Carbon possesses numerous ordered allotropes and intriguing amorphous structures. Disordered structures often exhibit magnetic moments arising from under‐coordinated atoms or specific atomic arrangements.^[^ ^40^ ^]^ Recent experiments and theoretical investigations suggest that magnetism in carbon nanostructures, including ferromagnetism and paramagnetism, is highly sensitive to the introduction of defects and symmetry breaking.^[^ ^41^ ^]^ Dangling bonds, which can carry magnetic moments, are also potential contributors to magnetic ordering.^[^ ^42^ ^]^ Magnetism and spin are intrinsically connected through the quantum properties of electrons. The intrinsic angular momentum of electrons, known as spin, produces a magnetic moment, resulting in each electron functioning as a miniature magnet.^[^ ^43^ ^]^ Non‐benzenoid rings, frequently identified as defects within graphene structures, can profoundly influence electronic properties through localized variations in strain and conjugation.^[^ ^44^ ^]^ Among the identified defects, pentagon defects in finite‐sized nanographene generate spin‐localized electronic states that are linked to increased magnetism, as demonstrated through scanning tunneling microscopy and spectroscopy techniques.^[^ ^45^ ^]^

**Figure 7:**
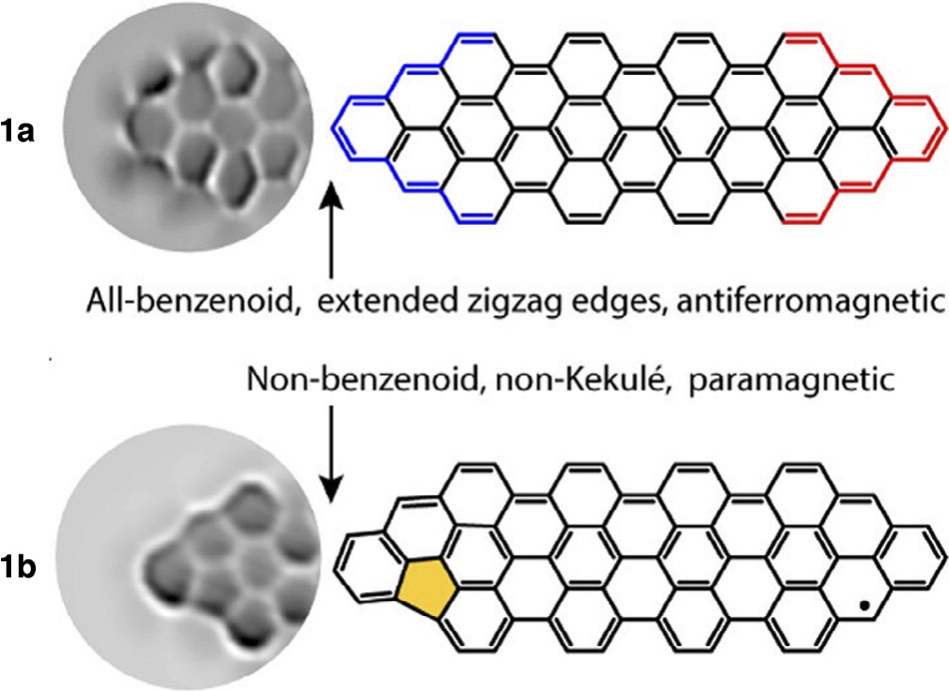
Structural characterization of 1a and 1b. Reproduced with permission.^[^ ^44^ ^]^ Copyright 2020, American Chemical Society.

The Fasel group successfully synthesized two distinct open‐shell nanographenes, designated as 1a and 1b (**Figure** **7**), through on‐surface reactions.^[^ ^44^ ^]^ Nanographene 1a exhibits an all‐benzenoid configuration characterized by zigzag edge‐rich termini, which accommodates low‐energy edge states and possesses a frontier electronic gap of 110 meV. In contrast, nanographene 1b presents a non‐benzenoid structure, incorporating a singular pentagonal ring within a benzenoid framework, thus categorizing it as a non‐Kekulé system, as demonstrated by the presence of a Kondo resonance. This research offers compelling evidence of all‐carbon magnetism and underscores the potential to induce and modulate magnetism in nanographenes via topological defects.

**Figure 8:**
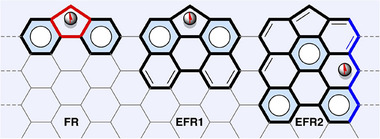
Pentagon defect and zigzag edge in nanographene with showing its major resonance structures. Reproduced with permission.^[^ ^45a^ ^]^ Copyright 2022, Wiley‐VCH.

The Sun group has indicated that as conjugation increases, there is a transition in the distribution of spin and charge from pentagon defects to zigzag edges.^[^ ^45a^ ^]^ As shown in **Figure** **8**, spin density calculations indicate a localized spin distribution at the pentagon site for the fluorenyl radical (FR) and extended fluorenyl radical 1 (EFR1), with a minimal spin presence on the zigzag edges. Conversely, for extended fluorenyl radical 2 (EFR2), the spin distribution predominantly shifts to the zigzag edges, exhibiting diminished significance at the pentagon site. This notable alteration in spin distribution can be elucidated through an analysis of the principal resonance structures featuring maximum Clar Sextets. In the cases of FR and EFR1, the radical situated at the pentagon site facilitates the formation of two Clar Sextets within the most stable resonance structures. In contrast, EFR2 preferentially accommodates radicals on the zigzag edges, allowing for the formation of three Clar Sextets, thereby rendering the pentagon‐centered structure less stable. This phenomenon highlights the recovery of Clar Sextets as a pivotal driving force for spin redistribution in extended conjugated systems.

### 2.2 Formation Mechanism

#### 2.2.1 Thermal‐Induced Transformation

In conventional methods, carbon materials are synthesized either by functionalizing carbon‐based raw materials (e.g., graphite) or through straightforward carbonization of substances with high carbon content, such as wood, coal, or shells.^[^ ^46^ ^]^ The conversion of non‐hexagonal rings generally necessitates adequate energy to surpass the activation barriers linked to atom migration and bond rotation. The Stone‐Wales (SW) transformation is a process that facilitates the conversion of four hexagonal structures into two pentagonal and two heptagonal configurations through the rotation of a pair of atoms by 90°. Nevertheless, this transformation is hindered by a significant kinetic barrier. This barrier effectively prevents the SW transformation from occurring at temperatures below 1000 °C.^[^ ^34^, ^47^ ^]^ Divacancy defects, which initially comprise two pentagons and one octagon, can evolve into a configuration featuring three pentagons and three heptagons. However, this transformation requires bond rotations that face activation barriers in the range of 5–6 eV.^[^ ^48^ ^]^ Electron beam irradiation is an effective technique for facilitating the evolution of non‐hexagonal rings. This phenomenon has been directly witnessed in graphene through the application of transmission electron microscopy (TEM).^[^ ^49^ ^]^

**Figure 9:**
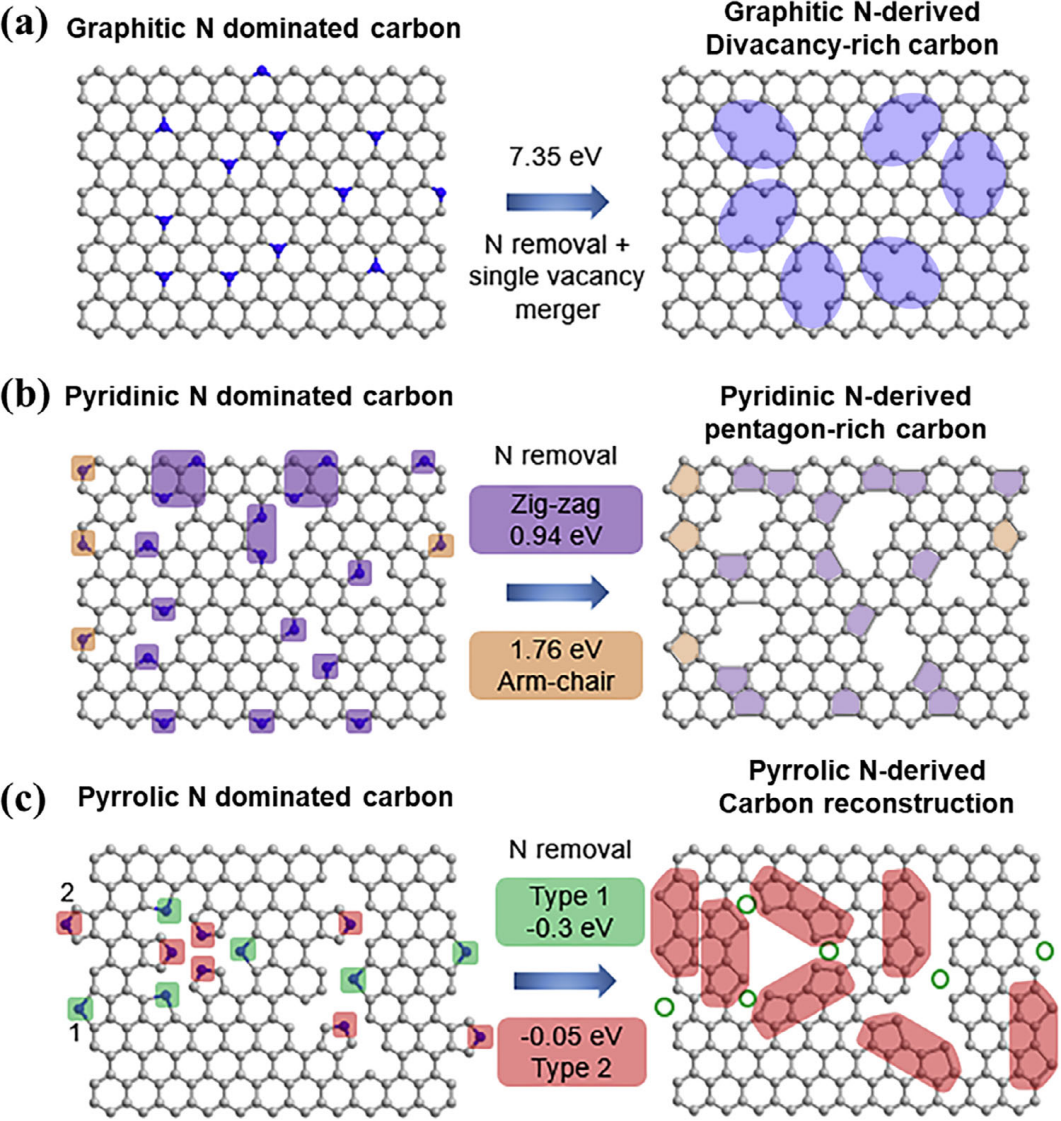
Computational simulation of specific N‐doping and removing process in different carbon models: a) schematic and formation energy calculation of transformation from graphitic N‐dominated carbon to divacancy‐rich carbon. b) Schematic and formation energy calculation of transformation from pyridinic‐N dominated carbon to pentagon‐rich carbon. c) Schematic and formation energy calculation of transformation from pyrrolic N dominated carbon to special carbon reconstruction. Reproduced with permission.^[^ ^50^ ^]^ Copyright 2020, Elsevier.

The formation and conversion of non‐hexagonal rings, including defects originated from atom migration and bond rotation, perform an essential function in adjusting the characteristics of carbon materials. By understanding the activation barriers and energy requirements for these transformations, researchers can explore innovative approaches to manipulate carbon structures. Besides, the incorporation and selective removal of heteroatoms like N offer promising pathways for controlled synthesis. This provides a theoretical foundation for the subsequent analysis of defect formation energies and the strategies for achieving specific configurations in carbon materials. The Zhou group employed DFT calculations to investigate the potential for defect‐controlled synthesis by selecting specific N‐doping configurations (**Figure** **9**). These arrangements consist of graphitic‐N, pyridinic‐N, and pyrrolic‐N.^[^ ^50^ ^]^ Energy formation assessments were conducted for each phase to validate theoretical viability. Specific topological carbon defects can be obtained by selectively removing particular N dopants. For example, the elimination of a graphitic‐N atom leads to the creation of a solitary vacancy. Nevertheless, solitary vacancies are unstable and tend to move and combine into divacancies (C585), which are more thermodynamically favorable. This change has an energy threshold of 7.35 eV. (Figure 9a), indicating that it necessitates considerable energy input, such as elevated‐temperature processing, to take place. In contrast, the elimination of pyridinic‐N and pyrrolic‐N atoms is considerably more achievable because of their notably reduced formation energy, generally under 2 eV (Figure 9b,c). Eliminating a pyridinic‐N atom, regardless of its position at a zigzag or armchair edge site, results in the formation of a pentagon defect at the edge (S‐C5). For pyrrolic‐N removal, DFT simulations reveal two possible outcomes: (i) Type 1, highlighted in green, leads to the restoration of a uniform hexagonal carbon framework; (ii) Type 2, highlighted in red, this leads to the formation of adjacent pentagon defects (A‐C5). These results demonstrate the feasibility of rationally designing starting carbon structures to attain particular N arrangements, along with their corresponding defect types. The insights provided by Figure 9 offer a theoretical basis for guiding experimental strategies in defect engineering.

#### 2.2.2 Bottom‐Up Method

Traditional pyrolysis technologies often struggle with uncontrollable structures and properties, resulting in suboptimal performance and poorly understood mechanisms in carbon materials. Nonetheless, considerable investigative advancements in the last ten years have resulted in the creation of a diverse array of carbon substances via thoughtfully engineered bottom‐up synthesis techniques.^[^ ^46^ ^]^ Among these, on‐surface synthesis, a bottom‐up method for forming covalent connections between molecular building units, has progressed considerably over the last ten years. Many reactions have been effectively accomplished and extensively examined on various surfaces, utilizing surface science methods in conjunction with theoretical computations.^[^ ^51^ ^]^ In this process, thermal activation is used to drive covalent bond dissociation and formation, enabling reactions such as dehalogenation, dehydrogenation, and radical cyclization. These methods allow for the programmable assembly of stable, conjugated carbon networks and the precise incorporation of controlled chemical functionalities. This approach allows for the creation of atomically precise and structurally uniform carbon‐based nanomaterials.^[^ ^52^ ^]^

**Figure 10:**
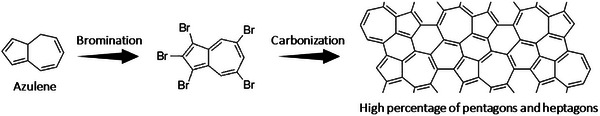
Carbon materials with high percentages of pentagons and heptagons were prepared from brominated Azu.^[^ ^53^ ^]^

**Figure 11:**
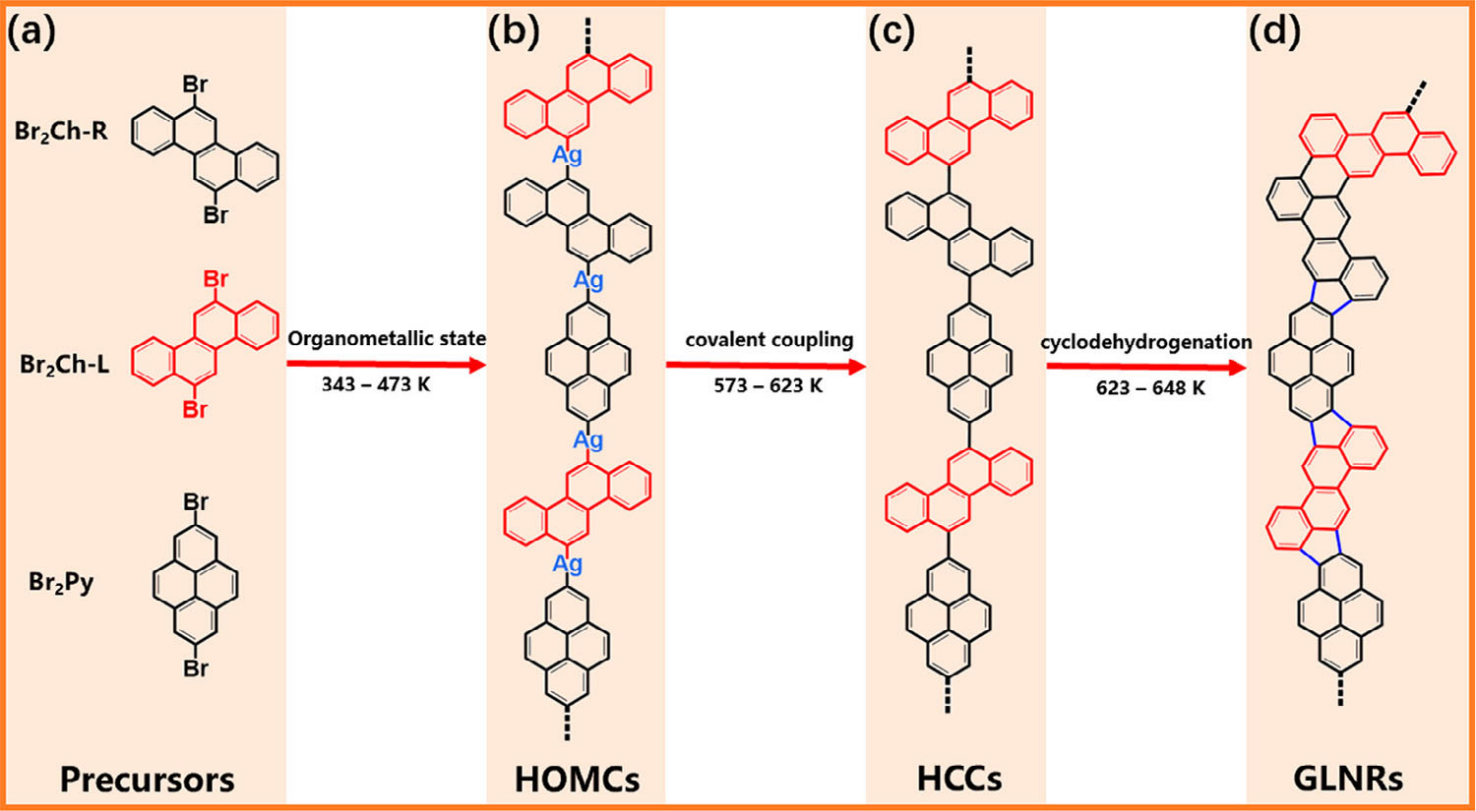
Reaction evolution process from two molecular precursors to pentagon‐incorporated GLNRs through progressive annealing: a) Br_2_Ch and Br_2_Py precursors, with the two enantiomers of the Br_2_Ch molecule marked with Br_2_Ch‐R (black) and Br_2_Ch‐L (red), respectively, b) hybrid organometallic chains, c) hybrid covalent‐coupling chains, and d) pentagon‐incorporated GLNRs. Reproduced with permission.^[^ ^56^ ^]^ Copyright 2023, American Chemical Society.

Yamada group disclosed a bottom‐up approach to producing carbon materials with elevated proportions of pentagons and heptagons.^[^ ^53^ ^]^ This method involves bromination and subsequent heat treatment at 873 K of azulene (abbreviation: Azu), which was composed of one pentagon and one heptagon without using catalysts (**Figure** **10**). Detailed analyses, including Raman spectroscopy (*I*_D_/*I*_G_ ratio), and X‐ray photoelectron spectroscopy (XPS) analysis (C1s peak broadening) of brominated and carbonized Azu indicate that the synthesized carbon materials comprise graphene‐like planes, with a high proportion of pentagons. Similar studies have utilized corannulene to produce carbon materials with a high proportion of pentagonal rings.^[^ ^54^ ^]^ The Fasel group announced the synthesis of 1D conjugated polymers featuring pentagons, as detected using noncontact atomic force microscopy (nc‐AFM).^[^ ^55^ ^]^ Similarly, the Song group demonstrated the synthesis of pentagon‐incorporated graphene‐like nanoribbons (GLNRs, **Figure** **11**d) through an on‐surface Ullmann coupling reaction and subsequent aromatic cyclodehydrogenation.^[^ ^56^ ^]^ Throughout this reaction process, surface adatoms are essential—not only in promoting the self‐organization of precursor mixtures but also in propelling the creation of intermediate hybrid organometallic chains (HOMCs) during annealing (illustrated in Figure 11b). Subsequent thermal processing (573–623 K) triggers the breakdown of organometallic connections, leading to the creation of C─C links, which results in hybrid covalent‐coupling chains (HCCs) between two precursors. (Figure 11c). At elevated temperatures, a cyclodehydrogenation process leads to the fabrication of GLNRs fused with pentagonal carbon rings, where the pentagon forms exclusively at the junction of two distinct precursors. The entire synthesis process on Ag(111) surfaces is tracked using scanning tunneling microscopy (STM) through stepwise annealing. This study highlights the critical role of aryl–metal interactions in directing self‐assembly and stabilizing the organometallic state. In addition, it provides important perspectives on the production of graphene nanoribbons and their derivatives directly on precious metal surfaces via Ullmann coupling and cyclodehydrogenation. This approach is especially encouraging for building heterojunction‐integrated nanoarchitectures utilizing various precursors.

Bottom‐up synthesis, which combines the rational design of molecular precursors with surface‐assisted methods to generate pentagon‐containing graphene nanostructures, is garnering increasing research attention in this field.^[^ ^33b^ ^]^

### 2.3 Characterization

Accurately characterizing structural defects in carbon materials remains a persistent challenge. While large‐scale characteristics such as surface area and porosity are comparatively simple to evaluate, providing definitive evidence for individual defects proves to be significantly more complex. To tackle this issue, various microscopic and spectroscopic techniques have been established, enabling more detailed investigations of structural imperfections, albeit often under stringent testing conditions. With the rapid advancements in developing characterization instruments, increasingly reliable and detailed insights into carbon defects are now within reach.^[^ ^19a^ ^]^

#### 2.3.1 Raman

**Figure 12:**
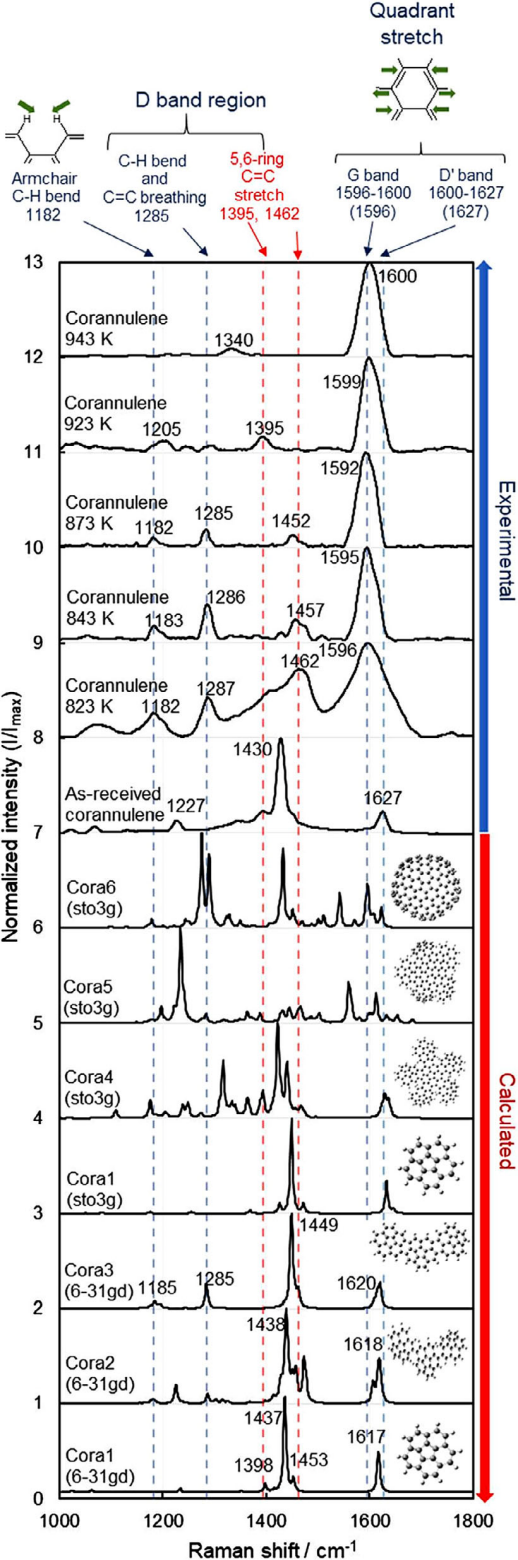
Calculated and experimental Raman spectra of as‐received and heated corannulene at different temperatures. The vibrations shown at the top of this figure are examples of vibrations. Other similar vibrations in different directions were also observed. Reproduced with permission.^[^ ^57^ ^]^ Copyright 2021, Springer‐Nature.

Raman spectroscopy is a commonly used method for analyzing graphitic carbon substances, particularly through the identification of two primary bands: the D and G bands, relating to defect content and graphitic domain size, respectively. To improve the interpretation of Raman data, efforts have been made to deconvolute these bands into four or five sub‐bands. A widely accepted explanation attributes the bands at ≈1500 cm⁻¹ to amorphous carbon.^[^ ^19a^ ^]^ **Figure** **12** illustrates the experimental Raman spectra of untreated and thermally processed corannulene, alongside the calculated Raman spectra of predicted carbonized corannulene structures.^[^ ^57^ ^]^ Experimental peaks for the untreated corannulene were detected at 1430 and 1627 cm⁻¹, relating to pentagonal structures and the D′ band (quadrant stretch), respectively. Upon carbonization, the peak associated with pentagons (1430 cm⁻¹) moved to the 1452–1462 cm⁻¹ range, while the D′ band (1627 cm⁻¹) transitioned to the 1592–1600 cm⁻¹ range, approaching the G band. The transition from 1430 to 1462 cm⁻¹ is connected to the creation of C═C coupling (Cora2) and the formation of new hexagons through dehydrogenation between corannulene molecules (Cora3–Cora6). These peaks, including components derived from pentagons, are commonly classified under the D band in Raman spectra of general carbon materials.

**Figure 13:**
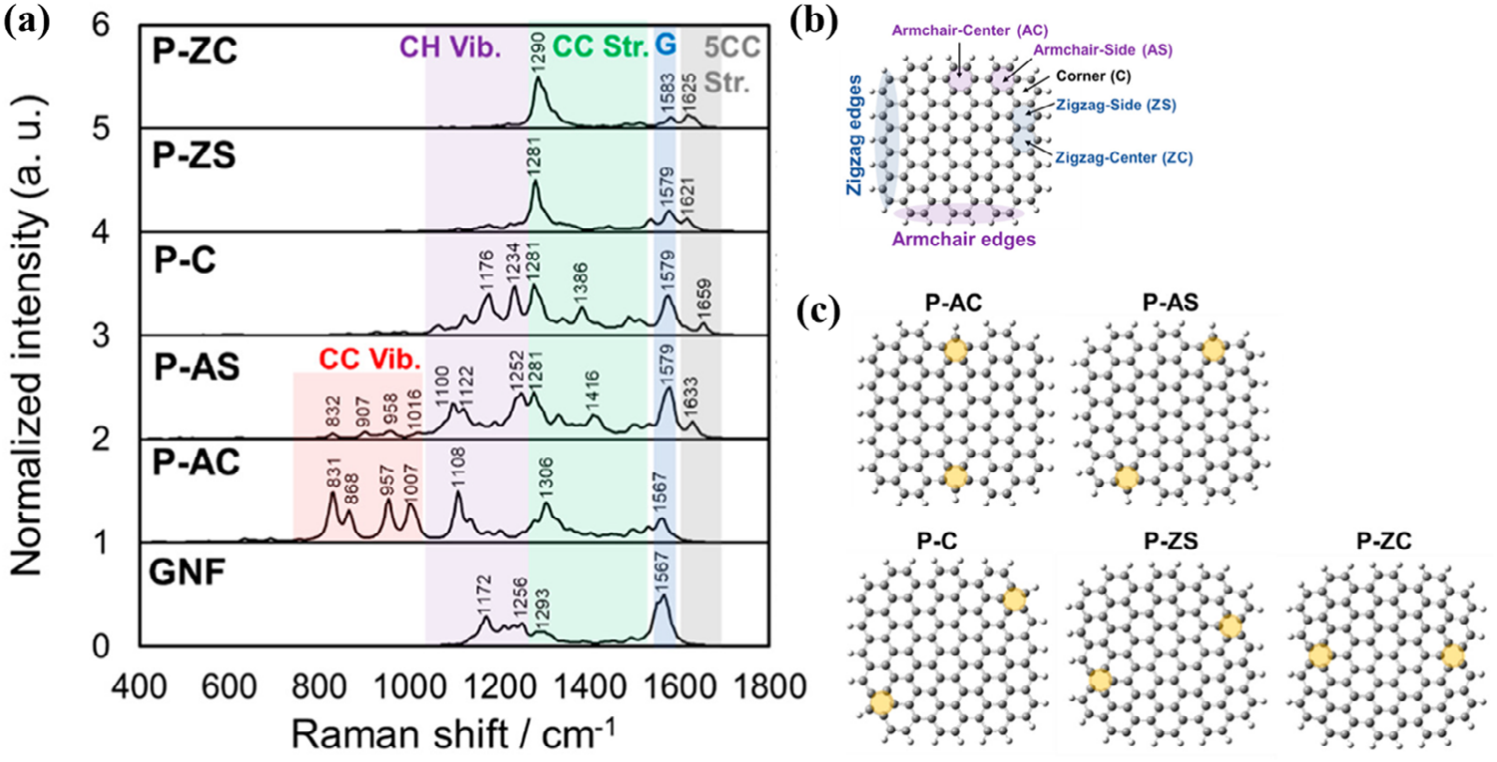
a) Simulated Raman spectra of graphene nanoflakes (GNFs) with pentagons. b) The basic structure of GNF (C_100_H_26_). c) Positions of the introduction of pentagons on zigzag and armchair edges in GNF. Reproduced with permission.^[^ ^58^ ^]^ Copyright 2021, American Chemical Society.

On the other hand, the simulated Raman spectra for structures with pentagons at the edges of graphene nanoflakes (GNFs) are displayed in **Figure** **13**a. Figure 13b,c demonstrates GNFs with and without pentagons introduced at five specific edge locations: armchair‐center (AC), armchair‐side (AS), corner (C), zigzag‐side (ZS), and zigzag‐center (ZC). The Raman spectrum of pristine GNF (Figure 13a) shows peaks at 1172 and 1256 cm⁻¹ (C─H bending vibrations), 1293 cm⁻¹ (C─C stretching), and 1567 cm⁻¹ (G band, C─C quadrant stretching). When pentagons were incorporated at the armchair‐center edge (P‐AC), four unique peaks associated with C─C vibrations emerged between 831 and 1007 cm⁻¹. Peaks in this range were also observed for P‐AS, but were absent in GNFs with pentagons on zigzag edges (P‐ZS and P‐ZC). C─H bending vibration peaks, evident in the pristine GNF, were detected in P‐AC, P‐AS, and P‐C but were negligible in P‐ZS and P‐ZC. Instead, GNFs with pentagons on zigzag edges exhibited a strong C─C stretching peak at ≈1290 cm⁻¹ and a pentagonal carbon stretching peak at ≈1630 cm⁻¹. To differentiate the type of edge where pentagons are presented, it is crucial to assess the presence of C─C stretching vibration peaks (800─1000 cm⁻¹) and C─H bending vibration peaks (1000–1200 cm⁻¹), along with pentagonal carbon peaks above 1600 cm⁻¹.^[^ ^58^ ^]^

#### 2.3.2 Electron Microscopy

As a relatively light element, carbon exhibits lower image contrast compared to heavier transition metals in electron microscopy, making optimal electron beam focusing particularly challenging. Furthermore, the potential side effects of electron beam exposure must be carefully addressed. Intense electron beams can harm specimens by causing combustion, while extended exposure may result in the graphitization of noncrystalline carbon, resulting in the elimination of defects. To address carbon polygons with exceptional accuracy, atomic resolution and aberration correction are essential. High‐resolution transmission electron microscopy (HRTEM) and aberration‐corrected high‐angle annular dark field scanning transmission electron microscopy (AC‐HAADF‐STEM) have emerged as standard methods for attaining these goals.^[^ ^19a^ ^]^

#### 2.3.3 X‐Ray Photoelectron Spectroscopy

**Figure 14:**
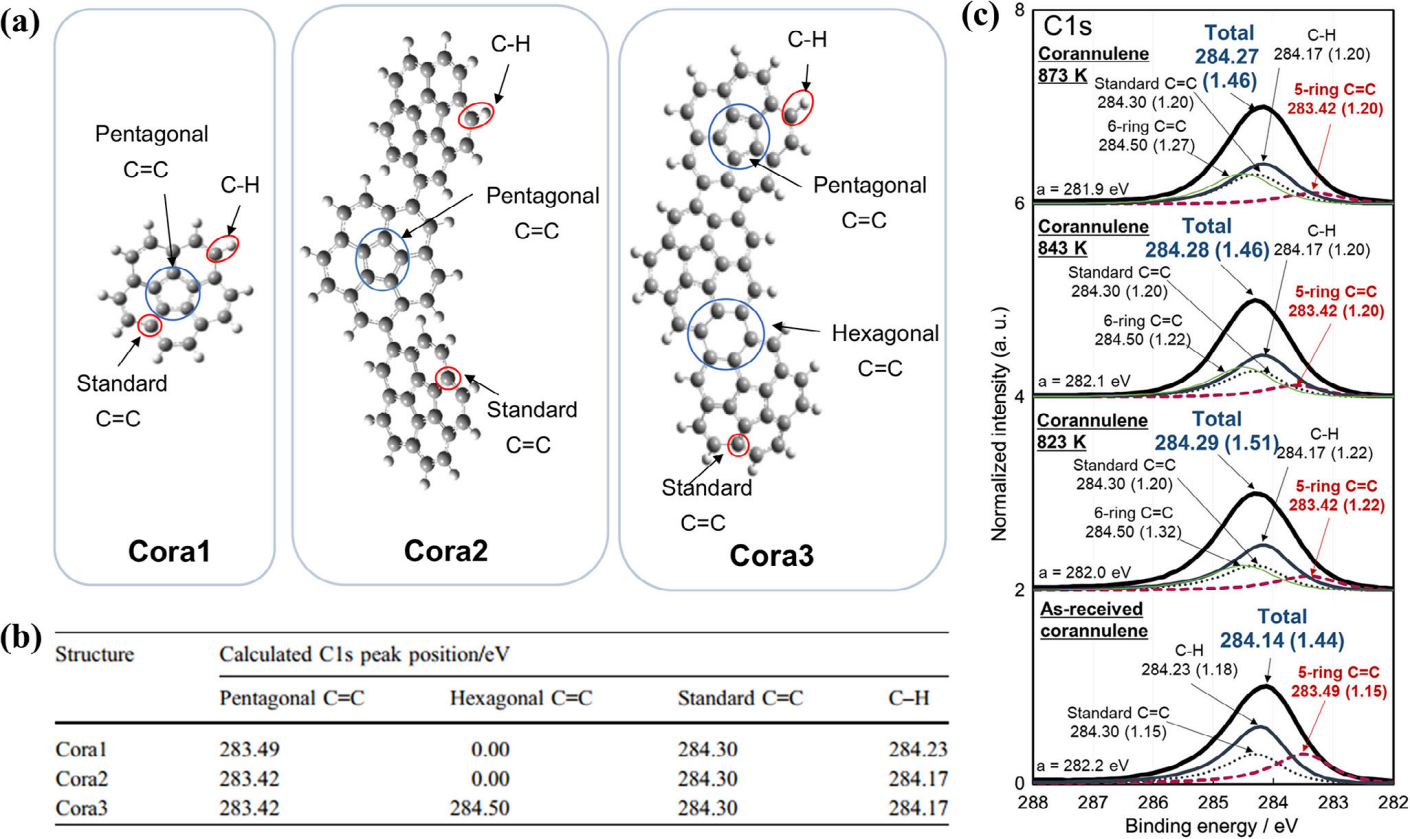
a) Structures of corannulene (Cora1), dehydrogenated corannulene (Cora2), and the further dehydrogenated corannulene (Cora3) used for assignments of C1s XPS spectra. b) Calculated peak positions of C1s XPS spectra for Cora1, Cora2, and Cora3. c) Experimental C1s XPS spectra of as‐received and heat‐treated corannulenes. “a” in this figure represents the C1s peak position before correction of peak tops using standard C═C. Reproduced with permission.^[^ ^57^ ^]^ Copyright 2021, Springer‐Nature.

XPS is an effective method for analyzing the bonding and hybridization of C atoms. **Figure** **14**a,b shows the calculated C1s XPS peak positions for a single corannulene molecule (Cora1), partially dehydrogenated corannulene (Cora2), and further dehydrogenated corannulene (Cora3).^[^ ^57^ ^]^ Heat treatment of corannulene molecules results in dehydrogenation and the formation of new hexagons, as seen in Cora2 and Cora3, which represent examples of carbonized corannulene with varying degrees of dehydrogenation. A comparison of the calculated peak positions for C ═C in pentagons, C═C in hexagons, and C─H in these three structures provides a framework for estimating experimental values at different stages of dehydrogenation. In Cora1, the C═C bonds in pentagons, which are fully surrounded by hexagons, exhibit a calculated binding energy of 283.49 eV—significantly lower than other characteristic peaks, such as C─H (284.23 eV) and standard C═C (284.30 eV) (Figure 14b). For Cora2 and Cora3, the calculated binding energy for C═C in pentagons shifts slightly to 283.42 eV. In Cora3, the calculated binding energy for C═C in the newly formed hexagons is 284.50 eV. These calculated peak positions for Cora1 serve as a reference for analyzing the experimental C1s XPS spectra of as‐received corannulene, while those for Cora3 can be used to interpret spectra of corannulene carbonized at 823–873 K (Figure 14c). Other studies have indicated that the peak position in the C1s spectrum of graphene nanoflakes can vary based on the location of the introduced pentagons.^[^ ^58^ ^]^ In the C1s spectra of the graphene nanoflake containing pentagons, the peak maxima are observed to range from 283.1 to 283.8 eV. The lower binding energy of the C─C(5) peak, compared to the C─C(6) peak, can be attributed to the electron‐withdrawing nature of the C─C(5) peak. These findings demonstrate that analyzing the full width at half maximum and peak shifts in C1s XPS spectra allows for estimating the number and spatial distribution of pentagons in graphene structures.^[^ ^59^ ^]^

## 3 Pentagon Defect in Carbon for Oxygen Reduction Reaction

Pentagonal defects in carbon materials represent a distinctive structural feature that offers a promising avenue for enhancing ORR catalysis. These defects disrupt the regular hexagonal lattice of sp^2^ carbon, introducing local curvature and strain that fundamentally affect the materials’ electronic character. As a result, pentagons create energetically favorable sites for O_2_ adsorption and activation, crucial for efficient ORR processes. Recent advancements in defect engineering have illuminated the critical role of pentagonal rings, with studies leveraging computational simulations and experimental analyses to demonstrate their exceptional catalytic potential. This section delves into the mechanisms by which pentagonal defects influence ORR activity, examining their behavior in both alkaline and acidic environments.

### 3.1 Activity in Alkaline Electrolyte

**Figure 15:**
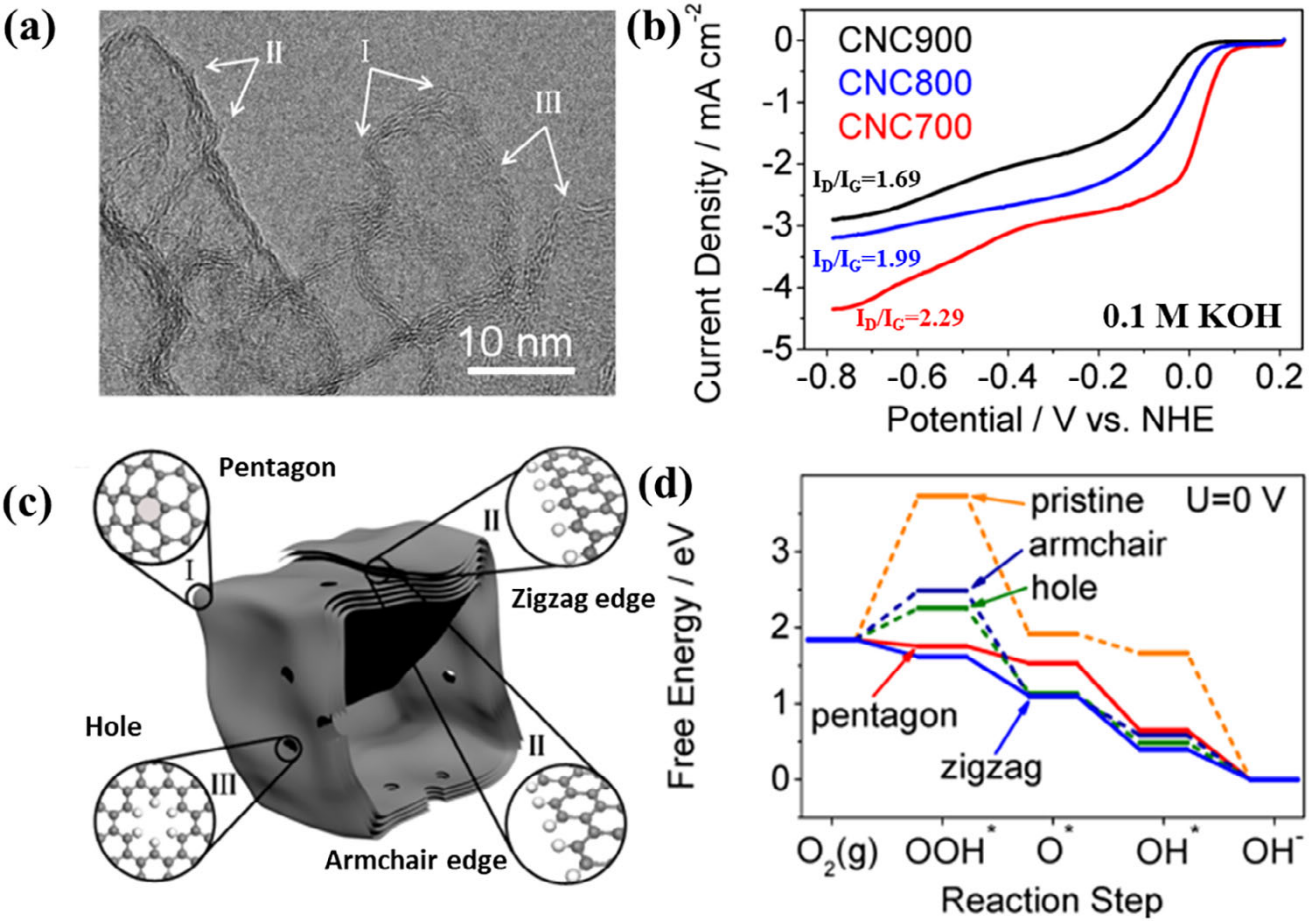
a) HRTEM image of CNC700. b) RDE curves of CNC700, CNC800, and CNC900 measured in O_2_‐saturated 0.1 m KOH at a scan rate of 10 mV s^−1^ with a rotating speed of 2500 rpm. c) Schematic structural characters of the carbon nanocages. I, II, and III represent three typical defective locations, i.e., the corner, the broken fringe, and the hole, respectively. d) Free energy diagrams for ORR activities of different defects. Reproduced under the terms of the CC‐BY license.^[^ ^60^ ^]^ Copyright 2015, American Chemical Society.

Hu group reported a carbon material with nanocage morphology, which was assumed with three types of defective structures.^[^ ^60^ ^]^ This nanocage was annealed under a series of temperatures to produce diversified defective characters. Among them, the CNC700 (**Figure** **15**a, treated at 700 °C) contains the largest amount of defects with the highest *I*_D_/*I*_G_ compared to CNC800 and CNC900. The corresponding ORR performance results in alkaline electrolytes (Figure 15b) show that the CNC700 also exhibits the best performance among the three samples. Although there is no direct evidence for the formation of the defects, it was proposed that the pentagon, as well as the zigzag edge defects (Figure 15c) embedded in carbon nanocages significantly contributes to high ORR activity. These findings are supported by DFT calculations (Figure 15d), which indicate that the ORR process is thermodynamically more favorable at pentagon and zigzag edge defect sites compared to other structural forms like armchair edges. This research highlights the importance of defects in carbon‐based catalysts, suggesting that creating and optimizing intrinsic defects like pentagons could lead to more efficient ORR catalysts.

**Figure 16:**
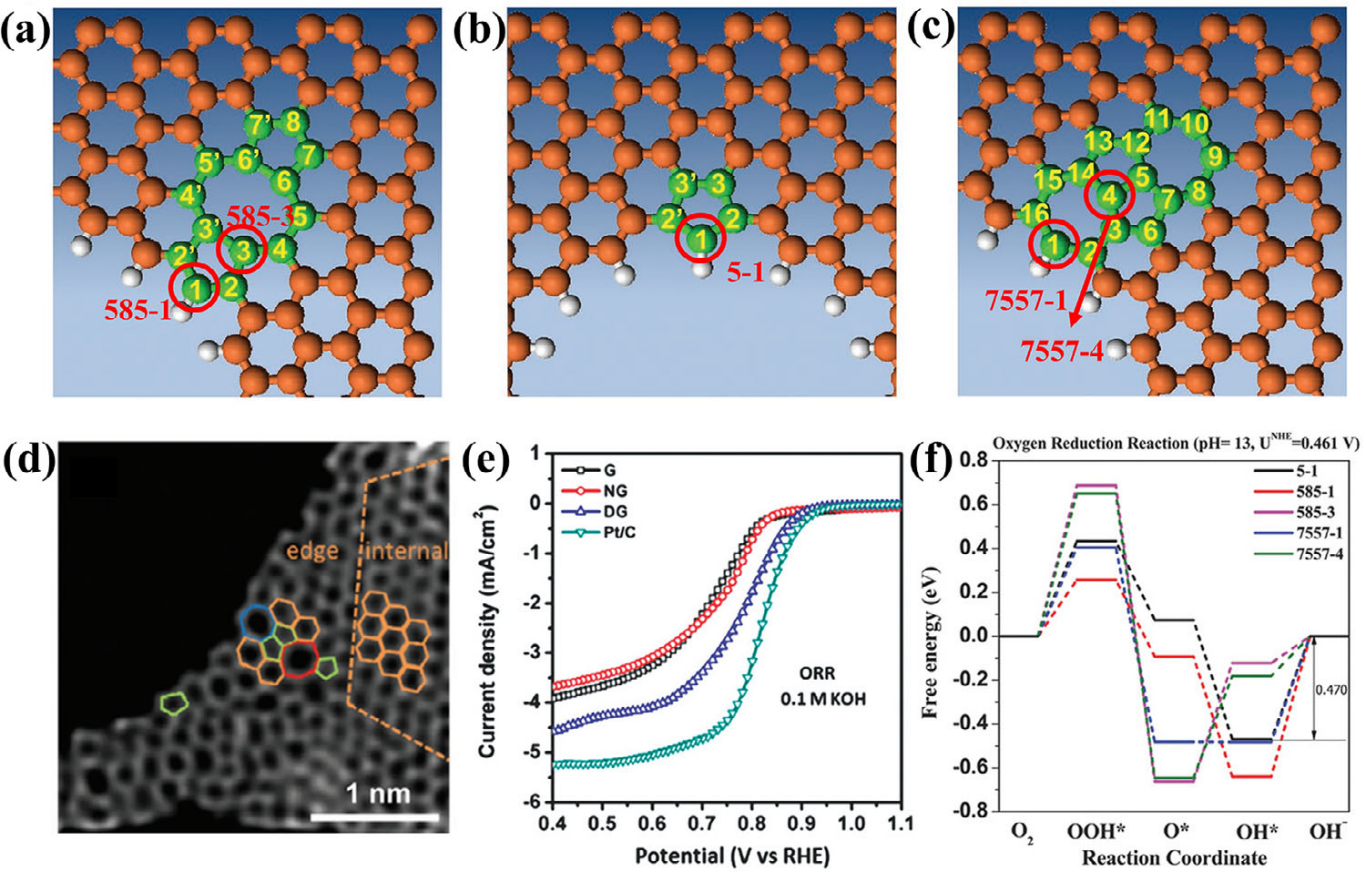
Schematic of a) 5‐8‐5 defect, b) edge pentagon, and c) 7‐55‐7 defect. Specific active sites were circled and labeled. d) HAADF image of DG with an acceleration voltage of 80 kV. Hexagons, pentagons, heptagons, and octagons were labeled in orange, green, blue, and red, respectively. e) Linear sweeping voltammetry curves for ORR of the pristine graphene, NG, and DG. f) Schematic energy profiles for the ORR pathway, on defective graphene in alkaline media. Reproduced with permission.^[^ ^61^ ^]^ Copyright 2016, Wiley‐VCH.

Yao group developed a defective graphene (DG) catalyst for the ORR.^[^ ^61^ ^]^ Various topological defects in the graphene lattice are illustrated in **Figure** **16**a–c, including (a) a 5‐8‐5 defect, (b) edge pentagons, and (c) a 7‐55‐7 defect. A HAADF image of DG (Figure 16d), highlights defective regions, where pentagonal defects were formed by eliminating N atoms from N‐doped graphene (NG) through heat treatment. This process rearranges C atoms, creating pentagons, heptagons, and octagons within the graphene lattice. The ORR activity of DG demonstrates an *E*_onset_ of 0.91 V versus RHE and a half‐wave potential (*E*_1/2_) of 0.76 V versus RHE in 0.1 m KOH electrolytes—significantly higher than that of NG and comparable to Pt/C (Figure 16e). DFT simulations confirm that pentagonal edge defects act as active sites, lowering activation energy and improving reaction kinetics. Five defect sites (5‐1, 585‐1, 585‐3, 7557‐1, and 7557‐4) exhibiting the highest catalytic activity have been identified. The free energy diagram (Figure 16f) shows that the most active site at a pH of 13 is identified as edge 5‐1, which exhibits the lowest activation barrier of 0.470 eV, followed by 7557‐1 (0.483 eV). This underscores the significant role of pentagonal defects in driving ORR performance.

**Figure 17:**
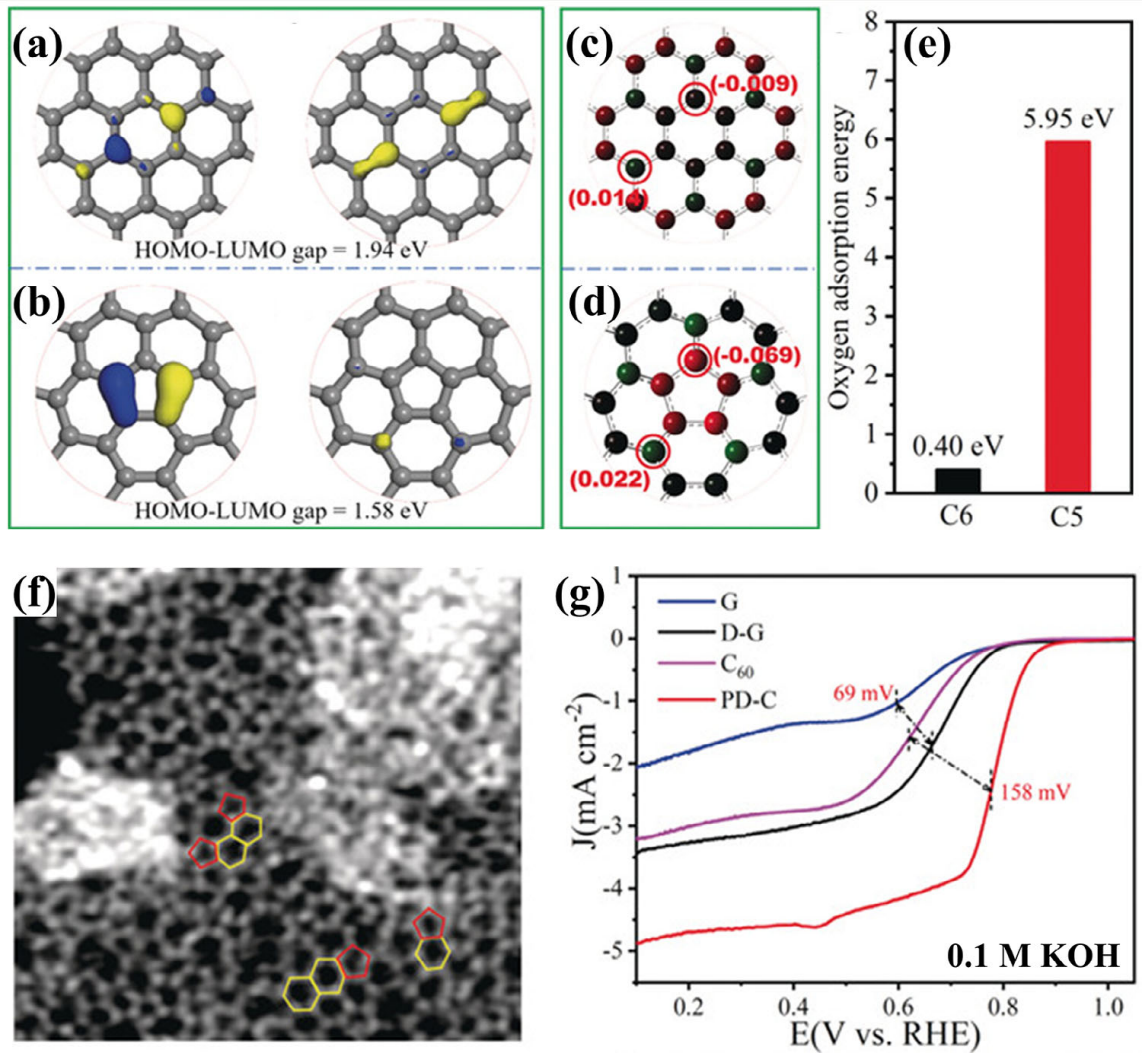
HOMO and LUMO distributions of a) C6 and b) C5. Charge densities of c) C6 and d) C5. e) O_2_ adsorption energies of C6 and C5. f) AC‐STEM image of PD‐C; g) LSV curves of G, D‐G, C_60_, and PD‐C catalysts. Reproduced with permission.^[^ ^62^ ^]^ Copyright 2019, Wiley‐VCH.

Mu group developed a carbon material characterized by a high density of pentagon defects (PD‐C) through the in situ etching of fullerene (C_60_).^[^ ^62^ ^]^ Theoretical calculations (**Figure** **17**a–e) reveal that pentagonal defects (C5) in carbon networks exhibit narrower HOMO‐LUMO energy gaps and higher charge densities compared to hexagonal structures (C6), making them more favorable for electron transfer. Moreover, C6 exhibits weak O_2_ adsorption (0.40 eV), while C5 shows much stronger adsorption (5.95 eV), confirming the potential of pentagonal defects as active ORR sites. An enlarged AC‐STEM image of PD‐C (Figure 17f) shows a carbon framework retaining both pentagons and hexagons. LSV curves (Figure 17g) indicate that PD‐C achieves an *E*_1/2_ of 0.78 V—158 mV higher than C_60_ and far outperforms defective graphene (D‐G). These results suggest that intrinsic pentagonal defects derived from C_60_ exhibit superior ORR performance compared to hexagons. Similarly, Lu group used melamine as the N source and dedope the N under 1000 °C to obtain increased pentagon defects to enhance the ORR activity.^[^ ^63^ ^]^

**Figure 18:**
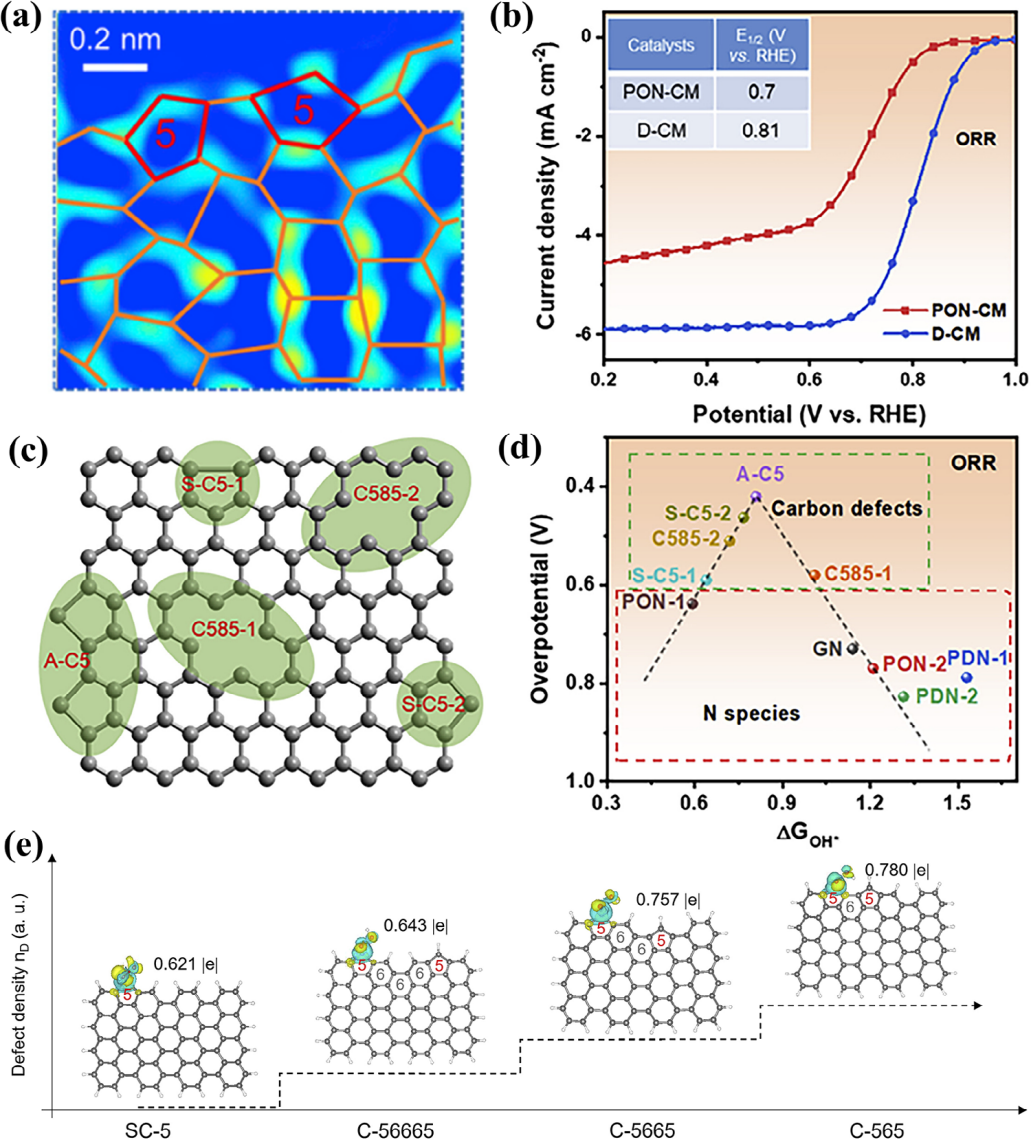
a) AC‐HAADF‐STEM images of D‐CM after fast Fourier transformation filtering. b) ORR performance of PON‐CM and D‐CM in 0.1 m KOH. c) The schematic of defect types for DFT calculations in (d). d) Volcano plots of overpotential versus adsorption energy of OH*, indicating carbon defects, especially the A‐C5 model, as more active sites than N species for ORR electrocatalysis. Reproduced with permission.^[^ ^50^ ^]^ Copyright 2020, Elsevier. e) The electron density corresponding to adsorbed OOH* on SC‐5, C‐56665, C‐5665, and C‐565 sites. Reproduced with permission.^[^ ^65^ ^]^ Copyright 2022, Elsevier.

Zhou group focused on synthesizing and exploring the role of adjacent pentagon defects in carbon materials.^[^ ^50^ ^]^ Using a zinc evaporation method, they developed an edge‐engineering approach to generate definitive defect configurations by converting specific N‐doping sites into adjacent pentagon defects (A‐C5). The defect‐rich carbon monolith with rich A‐C5 defect was obtained by removal of the pyrrolic‐N of pyrrolic‐N rich carbon monolith (POM‐CM). The AC‐HAADF‐STEM image of the D‐CM (**Figure** **18**a) highlights the presence of the A‐C5 defect. Figure 18b demonstrates the ORR performance of D‐CM compared to PON‐CM. The D‐CM achieved a higher *E*_1/2_ of 0.81 V, indicating superior catalytic performance compared to the PON‐CM. Furthermore, as illustrated in Figure 18c,d, the study identified that the A‐C5 defect demonstrated the highest catalytic activity for O_2_ reduction, surpassing other defect types such as divacancies (C585), separate pentagons (S‐C5), and N‐doping configurations. This highlights the critical role of adjacent pentagon defects in enhancing ORR performance. Unlike conventional defective carbon catalysts focusing on high defect density, the Xiao group developed a highly efficient ORR carbon catalyst featuring a graphitic structure embedded with pentagon defects. This was achieved through a simple dual molten salt treatment of polyperylene polymers, which simultaneously induces partial graphitization and defect formation. The resulting heteroatom‐free carbon nanosheets exhibit exceptional electrocatalytic ORR performance with a unique graphitic/defect (GDHfCN) structure.^[^ ^64^ ^]^ On the other hand, the Yao group emphasized that the precise quantitative control of defect density is crucial for improving catalytic performance.^[^ ^65^ ^]^ As shown in Figure 18e, the state of charge transfer between defect models and the OOH* intermediate is significantly influenced by incorporating neighboring defects. The transfer charge increased from 0.621 to 0.780 eV as the spacing between pentagon defects decreased, indicating an enhanced interaction between defects that promotes superior ORR activity.

**Figure 19:**
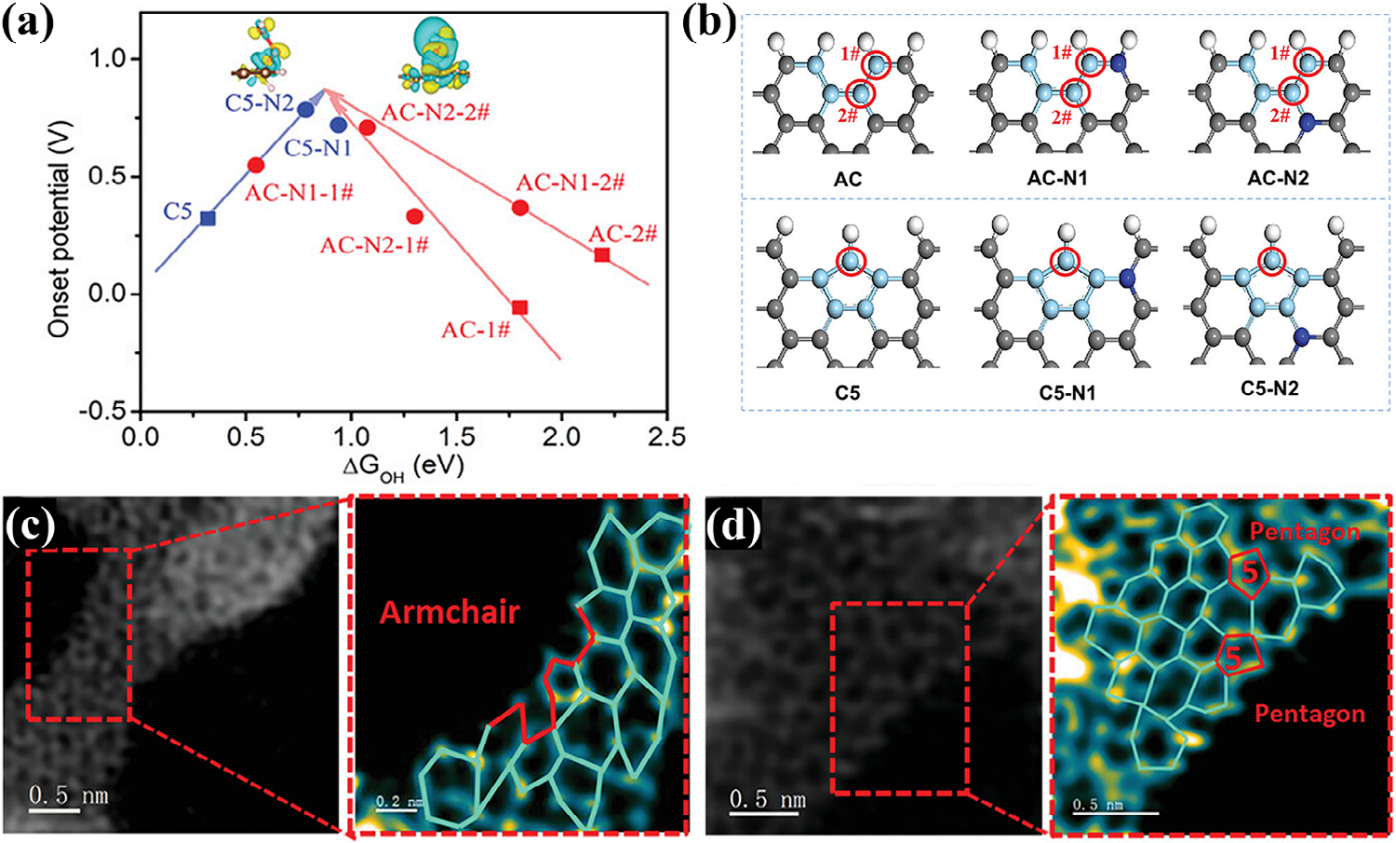
a) The Volcano diagram based on the Gibbs free energy of OH and the onset potential. The squares represent pure carbon defects, and the dots represent the structure after regulating the N element. b) The active site area of the six models. Armchair (AC) defect and five‐carbon (Pentagon, C5) defect, where each type of defect has two doped graphitic N configurations, N1 and N2, respectively. The red circle represents the active site. Light blue atoms represent defect C, gray represents other C, dark blue represents N, and white represents H. The AC=STEM image of c) armchair defects and d) pentagon defects of NDPC‐1000. The expanded image of the grayscale is transformed into a color gradient. Reproduced under the terms of the CC‐BY license.^[^ ^66^ ^]^ Copyright 2023, Wiley‐VCH.

Lin group developed a carbon catalyst (NDPC‐1000) with a graphitic‐N‐regulated defect structure, achieving outstanding ORR catalytic performance in alkaline media.^[^ ^66^ ^]^ DFT calculations (**Figure** **19**a,b) investigated the synergistic effects of graphitic‐N (G‐N) with armchair (AC) and pentagon (C5) defects. G‐N regulation was shown to enhance the activity of AC defects by increasing adsorption strength and optimizing C5 defect activity by facilitating intermediate desorption via electron donation. AC‐STEM images (Figure 19c,d) confirm the presence of AC and C5 defect types in NDPC‐1000, validating the theoretical predictions.

### 3.2 Activity in Acid Electrolyte

**Figure 20:**
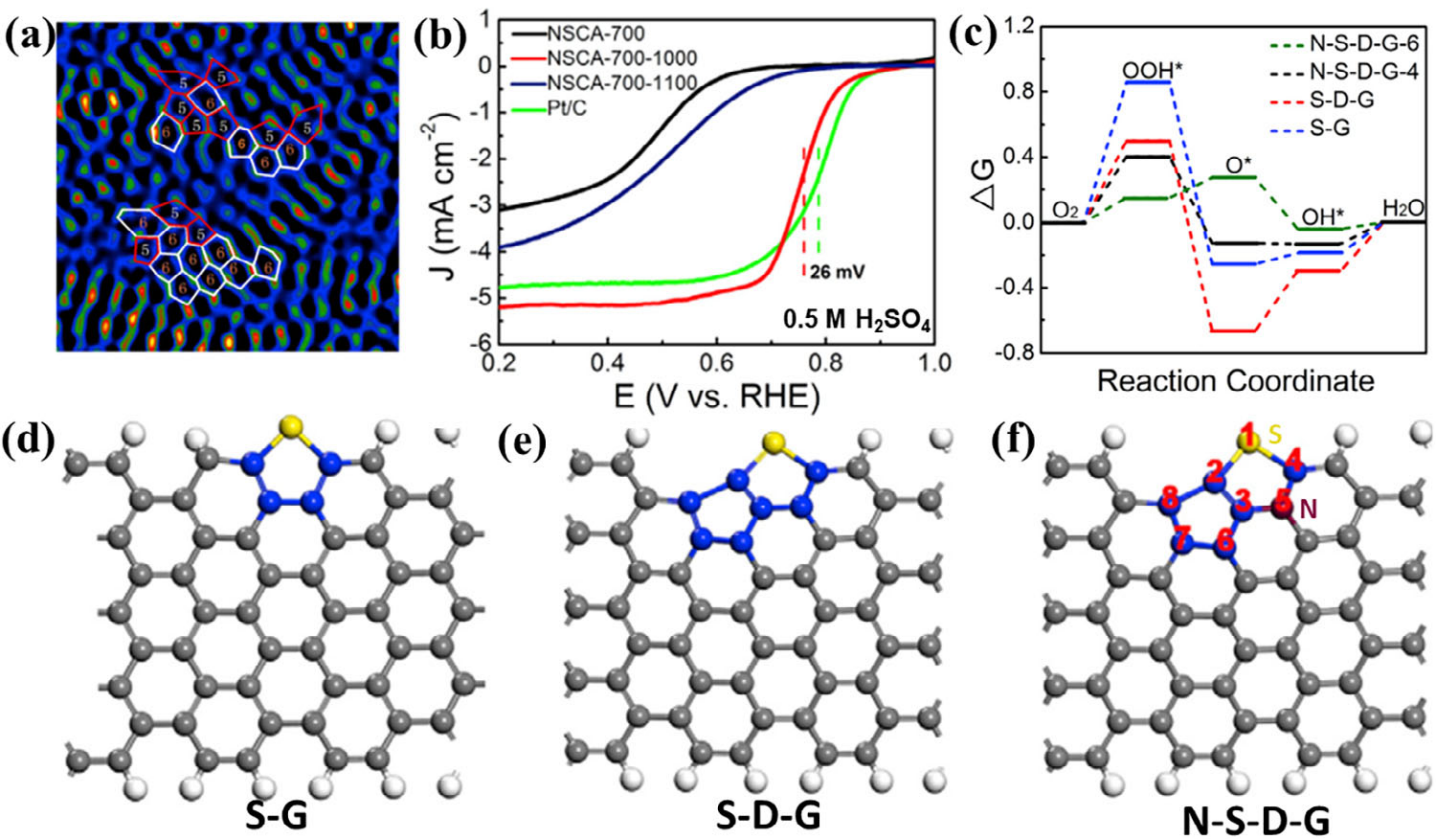
a) Filtered STEM images of NSCA‐700‐1000. b) LSV curves of NSCA‐700, NSCA‐700‐1000, NSCA‐700‐1100, and 20% Pt/C at 1600 rpm in 0.5 m H_2_SO_4_ solution. c) Free‐energy diagram for N‐S‐D‐G model, S‐D‐G, and S‐G at equilibrium potential. The optimized structures of d) S‐G, e) S‐D‐G, and f) N‐S‐D‐G model. Gray, white, yellow, and claret balls represent C, H, S, and N atoms; the blue atoms represent S‐ or N‐adjacent C atoms. Reproduced with permission.^[^ ^67^ ^]^ Copyright 2018, Elsevier.

Yao group reported the development of N‐S‐C coordination‐structured active sites, deriving from the synergy of edged thiophene sulfur (S), graphitic N, and pentagon defects, which brought the ORR performance closer to platinum‐based catalysts.^[^ ^67^ ^]^ The filtered STEM image of NSCA‐700‐1000 (**Figure** **20**a) presented a predominantly random carbon framework structure with observable pentagon defects and normal hexagons, resulting from the removal of doped N and S. The *E*_1/2_ of the NSCA‐700‐1000 reaches 0.76 V versus RHE in 0.5 m H_2_SO_4_, only 26 mV lower than that of commercial Pt/C catalysts (Figure 20b). DFT simulations (Figure 20c) were used to examine the model of S‐doped graphene (S‐G, Figure 20d), S‐defect graphene (S‐D‐G, Figure 20e) for SCA‐700‐1000, and N‐modified S‐defect graphene (N‐S‐D‐G, Figure 20f) for NSCA‐700‐1000. This combination of S and N atoms with pentagon defects also lowers the Gibbs free energy required for the ORR process, improving the overall reaction kinetics essential for high‐performance catalysis.

**Figure 21:**
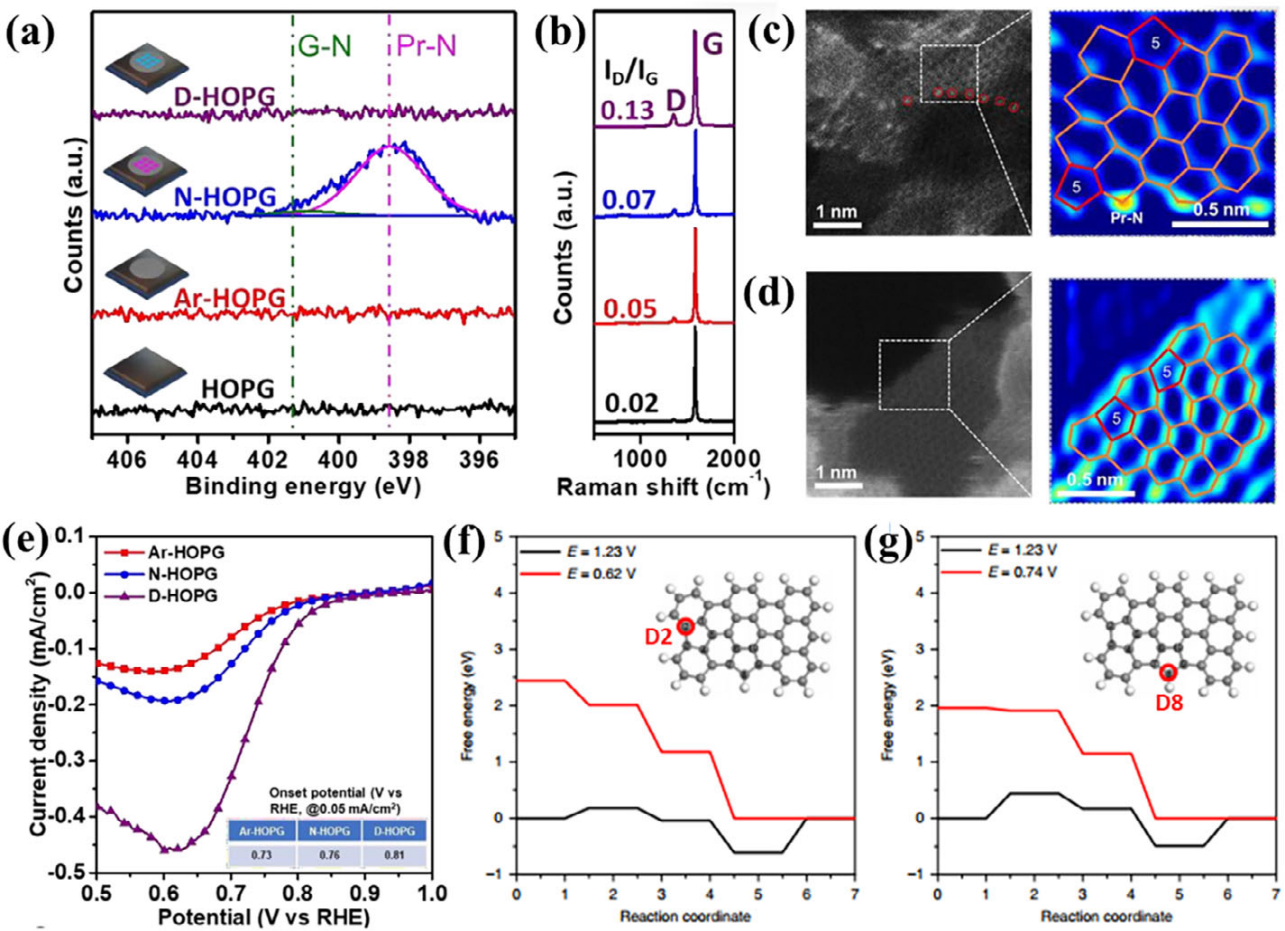
a) N 1s high‐resolution XPS spectra of HOPG, Ar‐HOPG, N‐HOPG, and D‐HOPG. b) Raman spectra of HOPG, Ar‐HOPG, N‐HOPG, and D‐HOPG. c) HAADF‐STEM image of N‐doped graphene (left, denoted as N‐G) and the partially zoomed‐in image of N‐G (right). The N atoms are marked with red circles. The grayscale is transformed into a color gradient. d) HAADF‐STEM image of derived defective graphene (left, denoted as D‐G) and the partially zoomed‐in image of D‐G (right). The grayscale is transformed into a color gradient. e) LSV curves of Ar‐HOPG, N‐HOPG, and D‐HOPG for ORR in 0.1 m H_2_SO_4_ solution. The correlated onset potentials are shown in the inset table. f,g) The energy profiles of D2 and D8 for ORR. The topological structures are inserted. Reproduced with permission.^[^ ^68^ ^]^ Copyright 2019, Springer‐Nature.

Xiao group demonstrated that pentagon defects at the edges are highly effective active sites for ORR, outperforming even N‐doped sites.^[^ ^68^ ^]^ **Figure** **21**a shows XPS data, comparing highly oriented pyrolytic graphite (HOPG), HOPG treated with argon (Ar‐HOPG), N‐doped HOPG (N‐HOPG), and defect‐engineered HOPG without N doping (D‐HOPG). Results showed that N‐HOPG is dominated by pyridinic N (Pr‐N) with trace graphitic N (G‐N), while N dopants are absent from D‐HOPG. Raman spectra (Figure 21b) showed an increased *I*_D_/*I*_G_ ratio in the order of HOPG < Ar‐HOPG < N‐HOPG < D‐HOPG, indicating new defects generated due to N removal. HAADF‐STEM images (Figure 21c,d) revealed N atoms (bright spots) in N‐G, but their absence was after high‐temperature treatment in D‐G, confirming the formation of pentagon defects. LSV curves for ORR in acidic media (Figure 21e) demonstrated that D‐HOPG exhibits the highest *E*_onset_ (0.81 V), outperforming both Ar‐HOPG and N‐HOPG. Free energy diagrams (Figure 21f,g) identified the zigzag pentagon defect (D8) as the most active ORR site, with an *E*_onset_ of 0.74 V.

**Figure 22:**
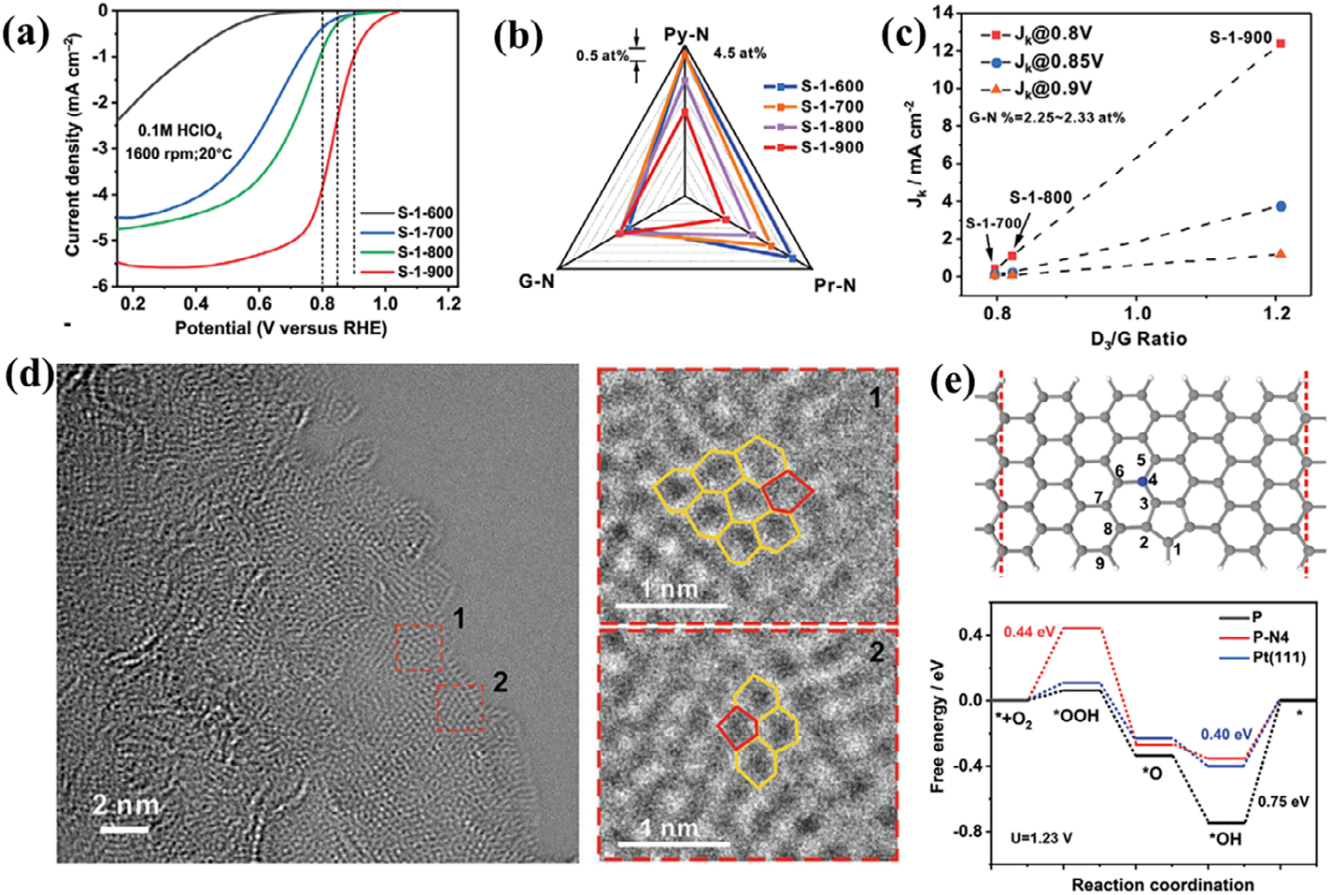
a) ORR result of S‐1 synthesized at different temperatures. b) Variety of N contents of S‐1 synthesized at different temperatures. c) Correlation of kinetic current densities at different electrode potentials and pentagon contents of S‐1 synthesized at different temperatures when the G‐N concentrations are at a similar level. d) ACTEM image of S‐1‐900. e) Enlarged images from the red dash squares from areas 1 and 2 in (d). e) N‐doped topological structure model and its corresponding free‐energy diagram at equilibrium potential 1.23 V. The one N‐substituted site in the pentagon structure is labeled with numbers 1–8, respectively, for instance, N‐substituted carbon at site‐4 (blue ball) is named P‐N4. The blue ball represents a N atom. Reproduced with permission.^[^ ^69^ ^]^ Copyright 2023, Wiley‐VCH.

Chen group reported a novel N‐doped carbon material, denoted as S‐1‐900, for ORR in 0.1 m HClO_4_, achieving an *E*_1/2_ of 0.81 V versus RHE and superior durability compared to commercial 20 wt% Pt/C catalysts (**Figure** **22**a).^[^ ^69^ ^]^ This material leverages graphitic N (GN) bonded pentagons in carbon to enhance intrinsic activity and increase active site density by expanding interlayer spacing. XPS (Figure 22b) and Raman (Figure 22c) analyses revealed a linear correlation between activity and the density of pentagons and adjacent GN atoms. AC‐TEM images (Figure 22d) confirmed abundant topological defects, including pentagons surrounded by hexagons at armchair edges. DFT simulations demonstrated that adjacent GN atoms considerably increase charge density at the C atoms in GN‐bonded pentagons, which act as active sites for ORR. As illustrated in Figure 22e, the energy barrier for OH* desorption decreases to 0.35 eV in the active site of P‐N4, in contrast to 0.75 eV in the active site of P. Similarly, the Li group reported a novel nitrogen‐doped porous carbon (PV/HPC) with abundant defect sites, including pentagons, edges, and vacancies, synthesized via a simple etching strategy, exhibiting excellent stability and catalytic performance in an acidic solution.^[^ ^70^ ^]^ In addition, the Lu group designed and synthesized a pentagonal defect‐rich N‐doped carbon nanomaterial (PD/N‐C) using C_60_ as a precursor with ammonia treatment. This material exhibits exceptional ORR activity, high 2e⁻ selectivity, and excellent stability in acidic electrolytes.^[^ ^71^ ^]^

Pentagonal defects in carbon materials are emerging as essential contributors to ORR catalysis, providing cost‐effective and sustainable alternatives to precious metal catalysts. Studies in both alkaline and acidic electrolytes highlight the superior catalytic activity of pentagon‐rich carbon structures compared to their hexagonal counterparts. **Tables** **1** and **2** provide a comprehensive comparison of various pentagon‐involved catalysts for ORR in acidic and alkaline, respectively, showcasing their synthesis methods, precursors, and performance metrics. The data emphasize the significant impact of pentagon defects on catalytic performance. The findings underscore the importance of defect engineering, highlighting how precise control over pentagon density and distribution can optimize ORR activity.

**Table 1:** Comparison of pentagon‐involved catalyst for ORR in acidic electrolyte.

Materials	Active site	Precursor	Synthesis method	Electrolyte	*E*_onset_	Refs.
N‐modified S‐defect carbon aerogel (NSCA‐700‐1000)	Graphitic N tuning of pentagon S‐defect	Carrageenan‐urea hydrogel	The aerogel was pyrolyzed at 700 °C in Ar flow and then annealed at 1000 °C in Ar flow	0.5 m H₂SO₄	0.85 V (vs RHE)	[67]
Defect highly oriented pyrolytic graphite (D‐HOPG)	Pentagon defects at the edges	Highly oriented pyrolytic graphite (HOPG)	Plasma method to form grooves in HOPG, ammonia annealing to dope N, and then the resulting sample was annealed at 1150 °C under N_2_ flow	0.1 m H₂SO₄	0.81 V (vs RHE)	[68]
S‐1‐900	Graphitic N‐bonded pentagons	Glycine	MgCl_2_ intercalation was used to exfoliate the nanosheet and form an in‐plane defect. the resulting sample was annealed at 900 °C under Ar flow	0.1 m HClO_4_	≈1.05 V (vs RHE)	[69]
Rich defects in nitrogen‐doped porous carbon (PV/HPC)	N‐doped porous carbon with pentagons, edge, and vacancy defects	ZIF‐8	Pyrolyzing the ZIF‐8‐derived NC with NH_4_Cl at 1000 °C under Ar flow	0.1 m HClO_4_	≈0.86 V (vs RHE)	[70]
Pentagonal defect‐rich nitrogen‐doped carbon nanomaterial (PD/N‐C)	Pentagon defect adjacent to the Gr‐N dopant	C_60_ and ammonia	Pre‐carbonizing C₆₀ at 1000 °C under Ar, followed by heat treatment at 1000 °C for 20 min and ammonia injection for 20 min at the same temperature	0.1 m HClO_4_	≈0.60 V (vs RHE)	[71]
Caged N‐doped Carbon (Caged‐NC‐1000)	Pentagon defects	C_60_ and ethylenimine	NaCl as a template; N‐doped C_60_ fragment was annealed at 1000 °C in N_2_ flow	0.1 m H₂SO₄	0.76 V (vs RHE)	^[^ ^72^ ^]^

**Table 2:** Comparison of pentagon‐involved catalyst for ORR in alkaline electrolyte.

Materials	Active site	Precursor	Synthesis method	Electrolyte	*E*_onset_	Refs.
Carbon nanocages (CNC700)	Pentagon and zigzag edge defects	Benzene	In situ MgO template method with growth temperatures of 700 °C in Ar flow	0.1 m KOH	≈0.11 V (vs NHE)	[60]
Defect graphene (DG)	Pentagonal defects at the edges	Graphene	N‐graphene was annealed at 1150 °C under N_2_ flow	0.1 m KOH	0.91 V (vs RHE)	[61]
Pentagon defects (PD‐C)	Pentagon defects	C_60_	KOH etching and then annealing at 900 °C in N_2_ flow	0.1 m KOH	≈0.85 V (vs RHE)	[62]
Defect carbon monolith (D‐CM)	Adjacent pentagon defects	Phenol, formaldehyde, and melamine	Zinc evaporation method to produce definitive pyrrolic‐N doped carbon and then annealing at 1150 °C in N_2_ flow	0.1 m KOH	≈0.95 V (vs RHE)	[50]
High defect density porous carbon (HDPC)	Dense pentagon defects	ZnO@ZIF‐7	Encapsulating zinc‐oxide quantum dots inside ZIF‐7; direct carbonize of ZnO@ZIF‐7 powders at 900 °C at N_2_ gas flow	0.1 m KOH	≈0.95 V (vs RHE)	[65]
N‐doped porous carbon (NDPC‐1000)	Graphitic N with armchair and pentagon defects	Adenine	MgCl_2_ as a hard template; the prepared sample was annealed at 1000 °C in Ar flow	0.1 m KOH	0.97 V (vs RHE)	[66]
Dedoped fullerene‐derived carbon (dFCMC)	Pentagon‐heptagon‐pentagon defect and the zigzag‐edged pentagons	C_60_ and melamine	Self‐assembling C₆₀ into 1D fullerene fibers (FFs), doping them with melamine at 900 °C, and then annealing at 1000 °C in Ar flow	0.1 m KOH	≈0.95 V (vs RHE)	[63]
Graphitic/defective heteroatom‐free carbon nanosheet (GDHfCN)	Pentagon defects and graphitic structures	Polyperylene and FeCl_2_/ZnCl_2_	Polyperylene was mixed with FeCl₃ and ZnCl₂, preheated at 400 °C, heated to 800 °C in N₂, and washed with 2.0 m HCl.	0.1 m KOH	≈0.96 V (vs RHE)	[64]

### 3.3 Stability of Pentagon Defect in Carbon

The stability of carbon‐based electrocatalysts is a critical factor in their practical applications in fuel cells. While significant progress has been made in engineering pentagon defect‐rich carbon materials for enhanced catalytic activity, their long‐term structural and electrochemical stability under operational conditions remains an area of growing interest.

Recent studies have investigated the stability of pentagonal defect‐rich carbon materials, primarily by comparing their performance with commercial Pt/C catalysts, demonstrating that pentagon defects can exhibit promising resistance to degradation under certain conditions.^[^ ^67^, ^70^, ^72^ ^]^ For instance, in Chen group's work,^[^ ^69^ ^]^ the S‐1‐900 exhibits excellent stability with a loss of only 11 mV in *E*_1/2_ after 10000th cycles from 0.6 to 1.0 V in 0.1 m HClO_4_ solution, which can be ascribed to the carbon oxidation in electrochemical cycles. The stability of S‐1‐900 is much better than Pt/C which shows 39 mV loss of *E*_1/2_ under the same test conditions. They further took a 100 h stability test at 0.8 V, the relative current density of S‐1‐900 decreases quickly at the first 20 h, it becomes much more stable in the next 80 h and maintains 61.4% relative current density. The HRTEM image of S‐1‐900 shows that the catalyst keeps a stable structure and morphology after catalytic reaction.

The degradation of carbon‐based catalysts in ORR primarily occurs through several key mechanisms, including oxidative attack by ORR intermediates, carbon corrosion, protonation‐induced structural instability, and micropore flooding.^[^ ^73^ ^]^ Oxidative degradation is particularly critical, as reactive oxygen species (ROS) and H_2_O_2_ generated during the ORR process can attack the carbon framework, leading to gradual loss of active sites and catalyst deactivation. In addition, under high electrochemical potential conditions, carbon corrosion results in CO_2_ formation, significantly diminishing catalyst durability. Furthermore, studies have shown that N‐doped carbon materials, while improving catalytic performance, may introduce additional degradation pathways.^[^ ^74^ ^]^ Despite these well‐known degradation pathways for carbon‐based materials, the specific degradation mechanisms of pentagonal defect‐rich carbon structures remain insufficiently understood. Currently, there is a lack of direct comparative stability data between pentagonal defect‐containing carbon materials and conventional graphitic structures. Future research should focus on systematically evaluating these materials under identical operational conditions to establish a deeper understanding of their durability.

### 3.4 Catalytic Mechanism of Pentagon Defects

Understanding the catalytic mechanism of the pentagon is essential for advancing their application in ORR catalysis.^[^ ^75^ ^]^ Unlike the conventional hexagonal lattice in sp^2^ carbon, pentagonal defects introduce significant structural irregularities, such as curvature and strain, which critically influence the electronic properties of the material. These irregularities alter charge distribution, enhance spin polarization, and facilitate the adsorption and activation of O_2_, making pentagonal defects highly active catalytic sites. However, the existing literature often falls short of providing a comprehensive analysis by focusing primarily on the adsorption energy of intermediates as a measure of catalytic performance. This approach overlooks critical factors such as spin polarization effects, the electronic interplay between pentagons and dopants,^[^ ^76^ ^]^ and the dynamic changes in curvature throughout the reaction process. A broader perspective is required to fully understand the catalytic origins of pentagonal defects.

This section will explore the fundamental principles linking pentagonal defects, spin polarization, and O_2_ absorption, emphasizing a more comprehensive understanding of their role in ORR. By digging into the interplay between curvature, spin, and electronic structure, this section aims to provide a comprehensive insight into why pentagonal defects serve as powerful catalytic sites in carbon materials.

**Figure 23:**
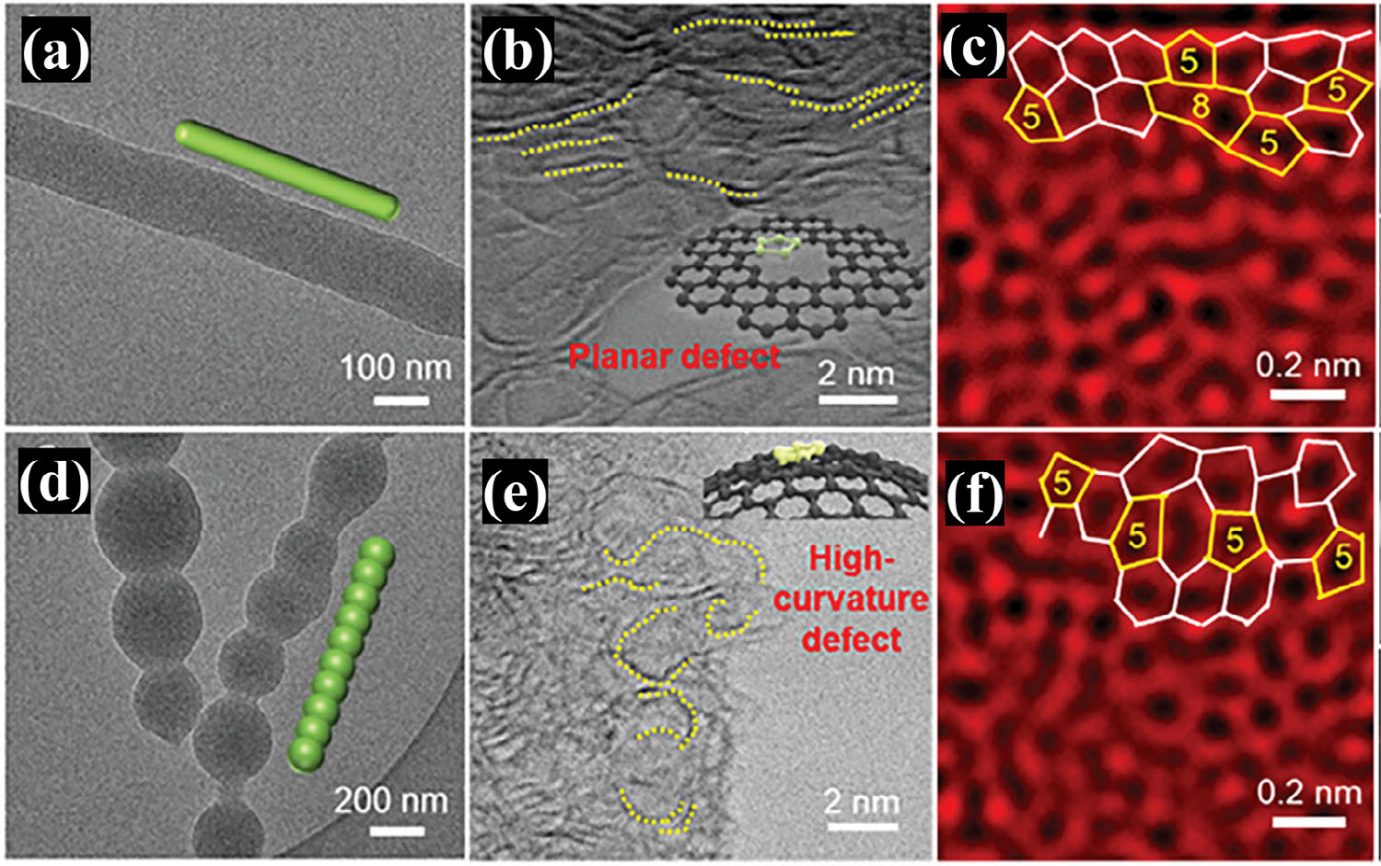
a,d) TEM images of LCDC and HCDC; the insets are corresponding morphology diagrams. b,e) HRTEM images of LCDC and HCDC; the insets are corresponding schematic atomic structures of carbon lattice. c,f) AC‐HAADF‐STEM images of LCDC and HCDC after fast Fourier transformation filtering. Reproduced with permission.^[^ ^77^ ^]^ Copyright 2024, Wiley‐VCH.

Wu group correlated the curvature of carbon defects to their ORR activity and demonstrated the superiority of highly curved topological defects in carbon.^[^ ^77^ ^]^ Theoretical calculations reveal that increasing curvature can regulate the electronic structure of edge pentagon defect, optimize the adsorption‐desorption process of reaction intermediates, and reduce the energy barrier for ORR. Experimentally, as shown in **Figure** **23**, using 1D phenol‐formaldehyde‐resin nanostructures with different surface morphologies as precursors to control the high‐temperature pyrolytic shrinkage process, they synthesize 1D carbon nanomaterials with rich topological defect sites of adjustable curvature. Performance testing shows that high‐curvature defective carbon (HCDC) displays a significantly improved ORR activity as compared to planar low‐curvature defective carbon (LCDC), with the kinetic current density at 0.8 V versus RHE increased by ≈17 times in alkaline electrolytes.

**Figure 24:**
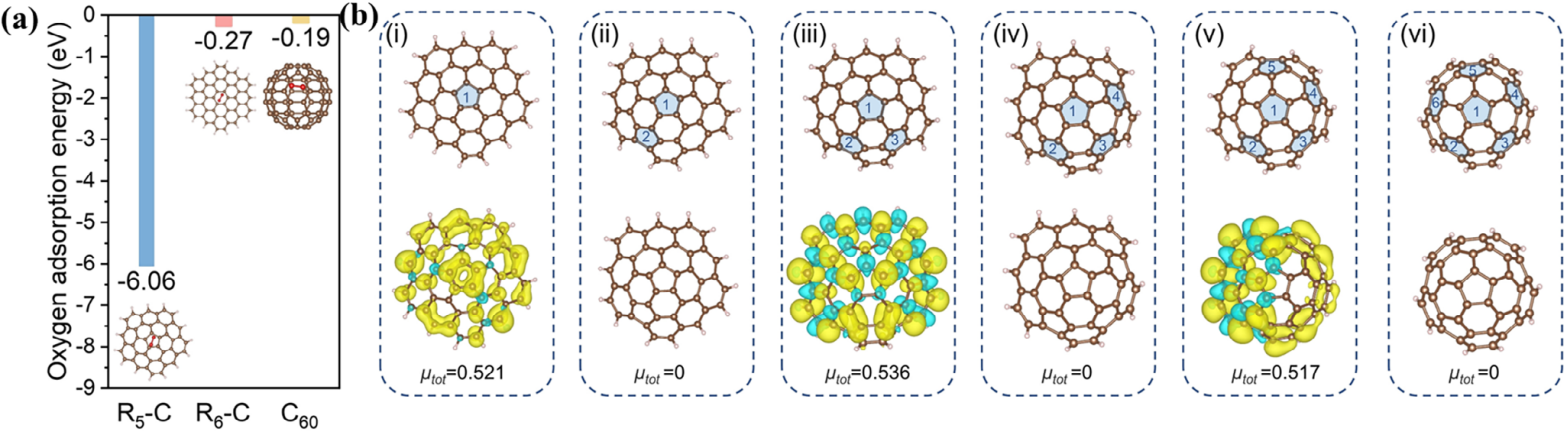
Adsorption property and spin density distribution of pentagons. a) DFT calculations based on VASP codes of the O_2_ adsorption energy of model R_6_‐C (hexagons) and model R_5_‐C (pentagons). b) DFT calculations based on VASP codes of the spin density distribution of different R_5_‐C models. (i) C_45_‐R_5_‐1 (one carbon pentagon ring among hexagons, 45C atoms in all, (ii) C_44_‐R_5_‐2 (two carbon pentagon rings among hexagons, 44 C atoms in all), (iii) C_43_‐R_5_‐3 (three carbon pentagon rings among hexagons, 43 C atoms in all), (iv) C_42_‐R_5_‐4 (four carbon pentagon rings among hexagons, 42 C atoms in all), (v) C_41_‐R_5_‐5 (five‐carbon pentagon rings among hexagons, 41 C atoms), and (vi) C_40_‐R_5_‐6 (six carbon pentagon rings among hexagons, 40 C atoms in all), respectively. Isosurface levels are 0.0055 eÅ^−3^. Reproduced with permission.^[^ ^78^ ^]^ Copyright 2025, Wiley‐VCH.

Cheetham group performed DFT calculations to understand catalytic active sites in the solid carbon spheres (**Figure** **24**).^[^ ^78^ ^]^ Models based on pure hexagons (marked as the R_6_‐C, is a small nanographene with pure hexagons) and pentagons (marked as the R_5_‐C, is a small nano defective graphene with one pentagon in substitution of the hexagon in the above R_6_‐C) in the basal plane were constructed for graphene and carbon sphere, in comparison to the C_60_, respectively. The adsorption energies of O_2_ on the R_6_‐C, R_5_‐C, and C_60_ models show that the R_6_‐C and C_60_ have weak O_2_ adsorption energies of −0.27 eV for R_6_‐C, and −0.19 eV for C_60_. In comparison, the R_5_‐C model presents a far larger O_2_ adsorption ability −6.06 eV, respectively. The adsorption energy on the R_5_‐C is much larger than that on the R_6_‐C (about 22 times) and on the C_60_ model (about 32 times). Therefore, it is more favorable for O_2_ adsorption on the pentagon of the model of R_5_‐C, rather than the hexagons of the mode of R_6_‐C or C_60_. To study the effect of the pentagon on the electron structure of carbon spheres, this work carried out the DFT calculations to obtain the spin density distribution by constructing a series of models (Figure 24b). DFT calculations on these modes indicate that an odd number of carbon pentagons in model molecules induces spin distribution (highly polarized spin configuration), while an even number of carbon pentagons have no spin distribution. Furthermore, the asymmetric models even with an even number of carbon pentagons also own polarized spin configurations, which are the catalytic activation sites. Thus, this group thinks curvature can greatly affect the electron structures of the spheres, which is caused by introducing positive curvature molecules, i.e., pentagon structures, into the carbon spheres. The kind of positive curvature pentagon defect can cause spin polarization and thus generate the catalytic active site, which is regarded as an intrinsic property of the asymmetric structure. They further pointed out that “curvature spin,” which can be viewed essentially as a superposition state of spin of bending σ‐spins and bending π‐spins, is ascribed to the symmetrical electronic structure broken.

**Figure 25:**
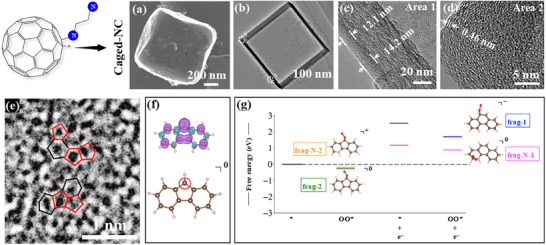
a–d) SEM and TEM images of Caged‐NC. e) Aberration‐corrected TEM images for Caged‐NC‐1100. f) Spin density distributions for frag‐2. The red circles indicate the atomic sites of maximum spin density in the molecules containing the pentagon. g) Free energy profiles of electron reduction and O_2_ adsorption processes for four fragment models: phenanthrene (frag‐1), a molecule with a pentagon (frag‐2), a molecule with an NH‐group (frag‐N‐1), and a molecule with an NH group and a pentagon (frag‐N‐2). Reproduced under the terms of the CC‐BY license.^[^ ^72^ ^]^ Copyright 2024, Wiley‐VCH.

The interaction between electron spin and O_2_ in carbon catalysts significantly influences the catalytic performance of the ORR. A promising approach to developing high‐performance catalysts involves introducing pentagon structures with spin electrons into graphitic carbons. Recently, Nakamura group presented the successful synthesis of cage‐like cubic carbon catalysts enriched with pentagon structures using pentagon‐containing C_60_ and a NaCl template.^[^ ^72^ ^]^ **Figure** **25**a–d shows the SEM and TEM images of the resulting Caged‐NC, which present the caged structures under the synthesis assisted by the NaCl template. A series of annealing experiments were conducted on Caged‐NC and Caged‐C (without N doping) to adjust the pentagon configuration, where the number of pentagons contained in the structure was increased by doping with N and annealing. The aberration‐corrected TEM images of Caged‐NC‐1100 (Figure 25e) provide insights into the structure, where Caged‐NC‐1100 showcases coupled pentagons, transforming during pyrolysis due to N doping, leading to pentagon defects. It was found that an increase in pentagon concentration corresponded to enhanced activity, indicating that the presence of additional pentagon defects contributes to the boost in catalytic activity. The higher density of the pentagon is believed to enhance the spin density. It can be seen from the density distribution of spin for frag‐2 (Figure 25f), that the spin density is highly localized on the vertices C atom of the pentagon (red circled C atom in Figure 25f). Furthermore, the DFT results indicate that the spin density localized on the pentagon enables O_2_ adsorption. (Figure 25g).

To sum up, pentagon defects enhance ORR catalysis by inducing curvature and electronic structure distortions, leading to localized spin polarization, which significantly impacts O₂ adsorption and activation. The presence of localized spin density at pentagonal sites further promotes efficient electron transfer, optimizing reaction kinetics and enhancing overall catalytic performance. The concept of spin electrons and the introduction of pentagon structures provide new design principles for carbon catalysts.

## 4 Conclusions

Pentagon‐containing carbon catalysts have recently emerged as promising candidates for ORR applications. Notably, these materials exhibit high catalytic activity even under acidic conditions, often outperforming N‐doped carbon catalysts. In addition to their superior activity, they demonstrate high durability and approach the ORR performance of Pt‐based catalysts, positioning them as strong candidates for future practical applications. The enhanced activity of these catalysts is attributed to the unique electronic structure induced by pentagon defects, which leads to spin formation. As oxygen adsorption, the initial step of ORR requires unpaired (radical) electrons, the presence of spin is a critical factor. It has been suggested that not only the presence but also the position of pentagon rings—particularly at zigzag edges—plays a crucial role in determining catalytic activity. These pentagon defects are closely associated with curvature‐induced strain, spin polarization, and localized charge density, all of which influence the ORR performance. It has been found that strain on the pentagon‐containing graphite surface in particular affects ORR activity.^[^ ^79^ ^]^ Pentagons have been introduced into carbon frameworks through various methods, including high‐temperature treatment, bottom‐up synthesis, and selective dopant removal. In addition, the synergistic effects of pentagonal defects in conjunction with heteroatom dopants such as nitrogen and sulfur have attracted considerable attention.

Based on the current understanding, future challenges in the field can be summarized as follows:

1) Defect engineering with atomic‐level precision

Optimization of the number, type, and spatial distribution of pentagonal defects is essential. While these defects enhance catalytic activity, excessive incorporation may compromise structural integrity. Furthermore, the catalytic behavior of pentagons located at different edge configurations (e.g., armchair vs zigzag) may vary, necessitating detailed investigations. A combination of experimental techniques and theoretical simulations will be indispensable for elucidating these structure‐activity relationships.

2) Development of highly active ORR catalysts

Strategic incorporation of pentagons into specific sites within the carbon matrix holds great promise. In addition to bottom‐up and thermal transformation approaches, plasma etching and template‐assisted growth have been proposed as effective methods. Plasma‐based techniques offer spatial control over defect formation, while template‐assisted synthesis enables the induction of pentagonal motifs within predefined architectures. Furthermore, the design of composite catalysts incorporating transition metals or other non‐metallic elements could open new avenues for performance enhancement.

3) Mechanistic elucidation of catalytic activity

Although spin and oxygen adsorption have been identified as key factors, the overall catalytic mechanism of pentagon‐containing carbon catalysts remains incompletely understood. Detailed experimental and theoretical studies—such as reaction pathway analysis and potential energy surface mapping—are required to provide mechanistic insights. Moreover, achieving high overall catalyst performance necessitates not only active local sites but also efficient O_2_ molecule transport and proton diffusion. The role of catalyst hydrophilicity and hydrophobicity in these processes warrants systematic investigation.

4) Performance optimization under practical operating conditions

To realize the practical deployment of these catalysts, performance validation under realistic conditions—such as membrane electrode assembly (MEA) testing—is critical. Optimizing gas diffusion and water management within the MEA requires integrated design at the micro‐, meso‐, and macro‐scale. Advances in this area will be instrumental in bridging the gap between fundamental research and commercial application.

## Conflict of Interest

The authors declare no conflict of interest.
